# Covalent aptamers: agents with promising therapeutic and diagnostic potential

**DOI:** 10.1039/d5cb00133a

**Published:** 2025-09-30

**Authors:** Savannah Albright, Jessica Boette, Mary Cacace, Alexander Deiters

**Affiliations:** a Department of Chemistry, University of Pittsburgh Pittsburgh PA 15260 USA deiters@pitt.edu

## Abstract

Small molecule- and antibody-based approaches have shown tremendous success in both therapeutic and diagnostic applications. Aptamers, which are engineered nucleic acid ligands for proteins, have not found similar broad applicability, potentially due to their susceptibility to nuclease-mediated degradation and short engagement times of their targets. One approach to mitigate these issues is the use of covalent aptamers. Here, the aptamer sequence is functionalized with an electrophilic motif, combining the high specificity of aptamer–protein binding with the ability to form a permanent covalent bond at nucleophilic residues on the target protein. These electrophilic motifs can be either non-cleavable, allowing for the formation of aptamer–protein conjugates, or cleavable, allowing for transfer of a payload onto the target protein. The chemical structures of these motifs define their functions which range from protein detection to targeted protein degradation. The covalent bond formed between the electrophile and a nucleophilic amino acid sidechain at the protein surface dramatically increases the engagement time and duration of action of the functional moiety. In this review, we summarize efforts in establishing, understanding, and applying the chemistry of covalent aptamers.

## Abstract 2

Covalent aptamers are a new class of oligonucleotide reagents. Covalent target engagement uniquely addresses limitations of traditional aptamers, resembling advances in covalent drug discovery.

## Graphical abstract

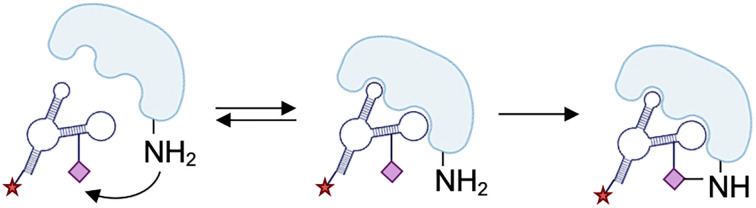

## 1. Introduction to aptamers

Aptamers are short, single-stranded oligonucleotides, typically 10–100 nucleotides in length, that are generated to bind proteins and small molecules.^1,2^ Their affinity and specificity for their targets rival that of antibodies, and can be attributed in large part to their ability to fold into unique three-dimensional conformations, including stems, loops, and quadruplexes.^3,4^ Aptamers utilize these three-dimensional poses to bind their target proteins over a large surface area through hydrogen bonding, electrostatic, and hydrophobic interactions.^5,6^ Despite similarities to antibodies, aptamers present distinct advantages over antibodies including (1) longer shelf lives and easy storage at room temperature, (2) facile and inexpensive generation *via* solid-phase synthesis, (3) amenability to a wide range of site-specific chemical modifications, (4) low batch-to-batch variability, and (5) low immunogenicity.^7^

**Fig. 1:**
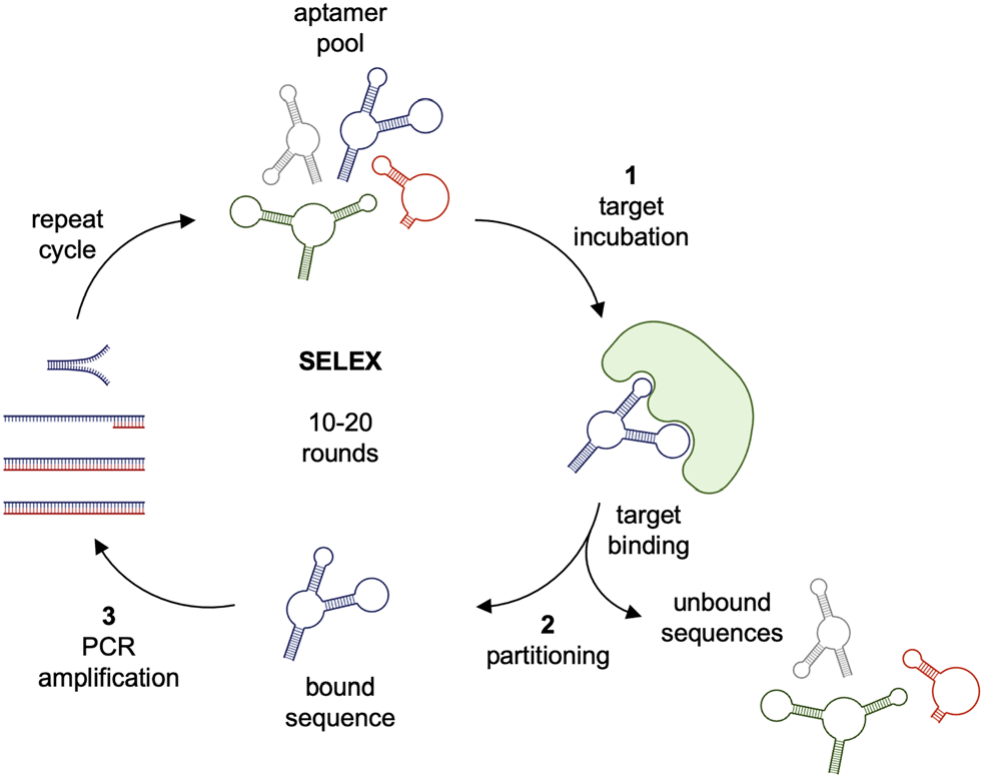
The SELEX cycle. A randomized aptamer pool is first incubated with an immobilized target protein. Unbound sequences are partitioned from the target-aptamer complex prior to eluting hit sequences. Bound sequences are amplified, and the cycle is repeated until significant sequence convergence is achieved. Select aptamer sequences are then resynthesized and evaluated for target binding.

While antibodies are developed through a lengthy and costly production process, aptamers are generated through an *in vitro* selection process termed SELEX (systemic evolution of ligands by exponential enrichment, Fig. 1) and therefore can, theoretically, be generated to target any protein.^1,2^ Aptamers are selected by (1) incubating a target protein or small molecule with a library of randomized oligonucleotide sequences, (2) partitioning unbound sequences from bound sequences, (3) amplifying the bound sequences, and (4) generating a new pool of ssDNA or RNA aptamers.^8^ After several rounds, potentially including rounds of negative selection with non-target proteins to enhance specificity, enriched aptamers are sequenced, individually synthesized, and evaluated. A number of alternative SELEX strategies have been developed including methods for the selection of aptamers targeting specific cells *in vitro* and in animals,^9–11^ approaches that cannot be conducted with antibodies. Next-generation sequencing has played a significant role in enhancing the SELEX process since it allows the tracking of sequence enrichment dynamics in real time, thereby dramatically reducing the number of necessary rounds. Furthermore, metrics like copy number and frequency shifts help prioritize candidate aptamers with strong binding properties through bioinformatics-driven identification of consensus sequences and structural motifs.^12,13^

**Fig. 2:**
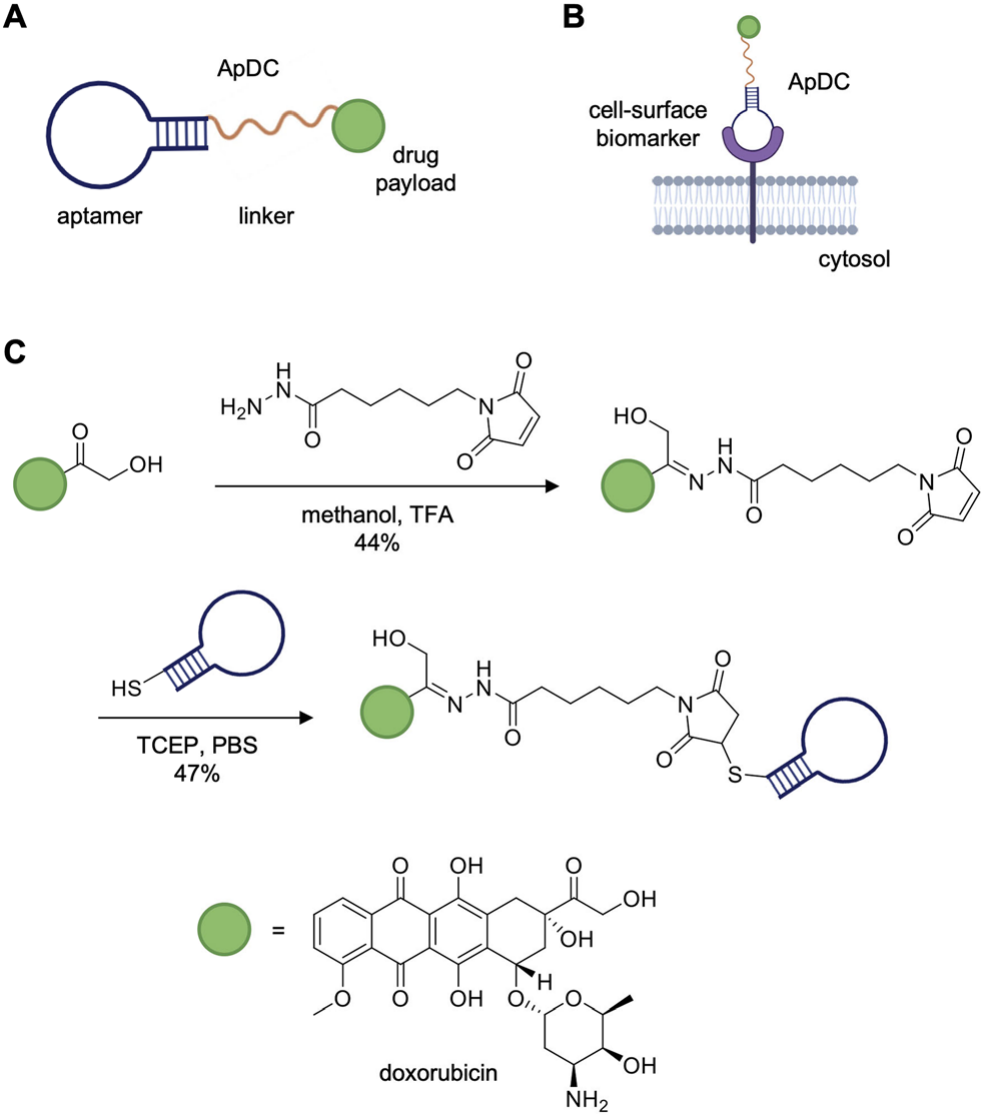
Aptamer–drug conjugates (ApDCs) for cell-specific drug delivery. (A) General design of an ApDC containing a drug payload (green) tethered to an aptamer (blue) *via* a linker (yellow). (B) The ApDC binds to its cell-surface target for the delivery of a drug payload to, for example, a cancer cell. (C) Synthetic scheme and yield of the sgc8c-doxorubicin ApDC targeting PTK7-positive cells. Parts of this figure were reproduced from ref. 23 with permission from John Wiley and Sons, copyright 2009.

Aptamers have been applied as both antagonists and agonists of biological processes, as well as diagnostic and targeted delivery agents.^7,14^ Two such agents, the RNA-based aptamers pegaptanib (Macugen)^15^ and avacincaptad pegol (Izervay)^16^ have been FDA-approved to treat age-related macular degeneration. As of 2024, there were eleven aptamers^17^ in clinical development to treat diseases such as macular degeneration,^18^ acute myeloid leukemia,^19^ and cardiovascular disease.^20^ Clinical trials of the nucleolin-targeting aptamer AS1411 demonstrated low toxicity and no signs of constitutive immune activation, but also showed low clinical efficacy requiring high dosages to be administered.^21^ As an alternative approach, aptamers have been covalently linked to therapeutic payloads opening up the potential for precise and specific delivery of drugs to target proteins. For instance, these drug-loaded aptamers have been equipped to bear cytotoxic small molecule generating aptamer–drug conjugates (ApDCs) similar to antibody–drug conjugates (ADCs, Fig. 2A).^22^ The aptamer plays an analogous role to that of the antibody in an ADC, guiding the small molecule drug to a cell-surface biomarker expressed on tumor cells, followed by internalization and drug release (Fig. 2B). One such example of an ApDC is the protein tyrosine kinase 7 (PTK7)-targeting aptamer, sgc8c, which was synthesized to carry the cytotoxic drug doxorubicin (sgc8c-Dox).^23^ The drug-aptamer conjugate was formed in two steps with moderate yields through attachment of doxorubicin to an *N*-(ε-maleimidocaproic acid) hydrazide linker and conjugation of the resulting maleimide-containing hydrazone to thiol-modified sgc8c (Fig. 2C). The ApDC reduced cell viability specifically in cell lines expressing PTK7 and exhibited an IC_50_ of 0.32 μM against CCRF-CEM cells, a PTK7-positive T-cell acute lymphoblastic leukemia line, which is a comparable to FDA-approved ADCs.^23,24^ Alternatively, an off-target ApDC was designed to test the specificity of aptamer-mediated drug delivery to CCRF-CEM cells. The TDO5 aptamer, specific for Ramos cells, a human Burkitt's lymphoma cell line, was conjugated to doxorubicin. PTK7-positive CCRF-CEM cells incubated with the TDO5-dox aptamer displayed no loss in viability or internalization of the off-target ApDC. Similarly, the sgc8c-dox aptamer had no effect on the PTK7-negative Ramos cells. Together, these studies demonstrate that sgc8c binding of PTK7 allowed for targeted drug delivery. Currently, there is an ongoing phase I clinical trial for AST-201, a GPC3-targeting aptamer with three incorporated gemcitabine drugs, targeting GPC3-positive advanced solid tumors.^25^ However, despite the success of aptamers in pre-clinical studies, these biomolecules lag behind antibodies in their clinical development.^26^

In addition to therapeutic advances, aptamers have gained traction as biosensors, diagnostic agents,^27–29^ and molecular imaging agents,^3^ including fluorescence imaging,^30^ magnetic resonance imaging,^30^ computerized topography,^31^ ultrasound imaging,^32^ and contrast imaging.^33^ For example, sgc8c, the aforementioned PTK7-targeting aptamer, was developed into a fluorescent probe through tethering the aptamer to a DNA sequence containing a fluorophore and a quencher.^33^ Only when bound to PTK7 on CCRF-CEM cell surfaces, the sensor underwent an induced conformational change resulting in fluorescence that was detected both *in vitro* and *in vivo*. Through similar nucleic acid engineering approaches, aptamers have been used in various systems for sensitive, early detection of viral infections,^34–40^ bacterial infections,^41–43^ and neoplastic diseases.^3,44–52^

While aptamers have comparable, in some cases superior, properties to antibodies, the *in vivo* potency of aptamers is limited due to their physiochemical characteristics. Their small size (as small as 4.7 kDa) and vulnerability to nuclease-mediated degradation negatively affect their pharmacokinetics, specifically metabolic stability and renal filtration.^6^ On the other hand, antibodies are unaffected by nucleases and, as large biomolecules of ∼145 kDa, are cleared more slowly than smaller aptamers. In addition, antibodies are protected from filtration and catabolism through a neonatal Fc-receptor (FcRn)-mediated recycling mechanism.^53^ It is important to note that the longer circulation time of antibodies can be beneficial or detrimental, based on the application.

The limitations of aptamers have been partially addressed through the incorporation of chemical modifications at the phosphate, sugar, and/or nucleobase level.^14^ Examples of such modifications include the incorporation of polyethylene glycols (PEGs), phosphorothioate linkages, 2′-fluoro, 2′-amino, or 2′-*O*-methyl modifications, and inverted thymidines for 3′-end capping.^6,54^ Alternatively, xeno-nucleic acids (XNAs) have been developed in which the canonical ring of DNA and RNA is replaced by various artificial motifs, including hexitol nucleic acids (HNA), α-(l)-threose nucleic acids (TNA), locked nucleic acids (LNA), and 2′-fluoro-nucleic acids (FNA). These modified sugars provide enhanced nuclease resistance and an expanded chemical repertoire for aptamers.^55,56^ Six of the eleven aptamers currently in clinical development as well as the two approved aptamer therapeutics, pegaptanib and acacinaptad pegol, all include some form of chemical modification to enhance their pharmacokinetic properties.

Previous studies have shown that the off-rates of aptamers are 10-fold higher than those of antibodies.^57^ These high off-rates can possibly be attributed to their confirmational lability as well as the SELEX process itself. SomaLogic developed slow off-rate modified aptamers (SOMAmers) to counteract the fast off-rates of aptamers. SOMAmers are designed to contain one or two^58^ nucleotides modified with groups mimicking amino acid side chains, *e.g.*, benzyl, naphthyl, tryptamino, or isobutyl moieties. These non-canonical side chains are capable of interacting with hydrophobic residues on their target proteins, thereby enhancing the stability of the interaction and consequently on-target residence time.^59,60^ This approach has been used to develop aptamers with enhanced specificity for thousands of protein targets and provides the foundation for a commercially available multiplexed, aptamer-based proteomic assay for biomarker identification.^60–62^

One emerging technology that holds promise in addressing rapid clearance and short target engagement times, while simultaneous enabling new applications, are “covalent aptamers”. Here, covalent aptamers are defined as any aptamers that have been functionalized with an electrophilic group suitable for attack by nucleophilic amino acid residues resulting in covalent bond formation. The aptamer acts as a ligand, binding the surface of the target protein and correctly positioning the electrophile such that proximity-driven covalent bond formation occurs. The earliest report of a covalent aptamer was by the Repine group in 1995, where a covalent active site inhibitor, based on a diphenyl phosphonate electrophile, of the protease the human neutrophil elastase (hNE) was used as an anchoring point for SELEX. The RNA aptamer library was attached to the covalent inhibitor through RNA:DNA duplex formation,^63^ and aptamers were identified that showed increased specificity and potency compared to the small molecule inhibitor alone. A rat lung perfusion model was used to assess *ex vivo* performance of the aptamer-small-molecule conjugate and provided proof-of-concept evidence that targeted covalent inhibitors against hNE could be effective in treating inflammatory lung diseases. Lungs treated with the covalent aptamer showed significantly less structural injury compared to untreated controls. This report was followed by a study by the Zichi group in 1997, where aptamers specific for the cell adhesion glycoprotein l-selectin were modified with a 3′-aldehyde group capable of crosslinking to proximal lysine residues through a reductive amination in order to characterize the protein/aptamer complex.^64^

In the design of covalent aptamers, electrophile modification should be chosen appropriately to avoid undesired intramolecular reactions, as work by several labs has shown the possibility for alkylation of the N7-position of guanosine and acylation of the 2′-hydroxyl group of RNA transcripts.^65–69^ Of note, recent reports by the Micura group describe efficient and selective alkylation of the N7 position in RNA through the use of a mesyl electrophile, thus indicating that the choice of electrophile is key for covalent aptamer assembly and successful protein labeling.

Ideally, a covalent aptamer in a therapeutic setting would have similar desirable characteristics to those of small-molecule covalent inhibitors, namely high target specificity, high affinity, and favorable pharmacokinetics. Covalent target engagement can allow for higher potency, lower dose, and extended duration of action compared to non-covalent inhibitors, possibly decreasing the chance for off-target engagement. Additionally, arguments have been made that irreversible action of covalent bond formation and slow protein turnover reduces off-target effects *in vivo* through prolonged, single-event target engagement, as free aptamer is systemically cleared.^70^ Complete degradation will release a free electrophile, which, without a proper targeting motif, will be at a concentration too low to have a discernable effect. Due to the need for a proximal nucleophilic amino acid for the covalent interaction to occur, covalent aptamers could show increased specificity compared to their non-covalent counterparts depending on the binding interface of the target protein.^70^ However, concerns remain regarding the safety profile of covalent therapeutics. Covalent drugs can elicit undesired immune responses through hapten formation, as observed with β-lactam antibiotics, where the covalent modification of host proteins can trigger allergic reactions.^71^ Additionally, the irreversible nature of covalent bond formation may capture transient, non-specific interactions that would normally dissociate rapidly, potentially leading to off-target protein modification and associated toxicity. Here, the recent developments and applications in the field of covalent aptamers will be discussed, exploring their underlying chemistry and mechanisms of covalent bond formation. We will examine their potential as therapeutic agents, including their advantages in terms of target engagement and pharmacokinetics, as well as their emerging applications as diagnostic tools.

## 2. Development of covalent aptamers and utilized electrophiles

**Fig. 3:**
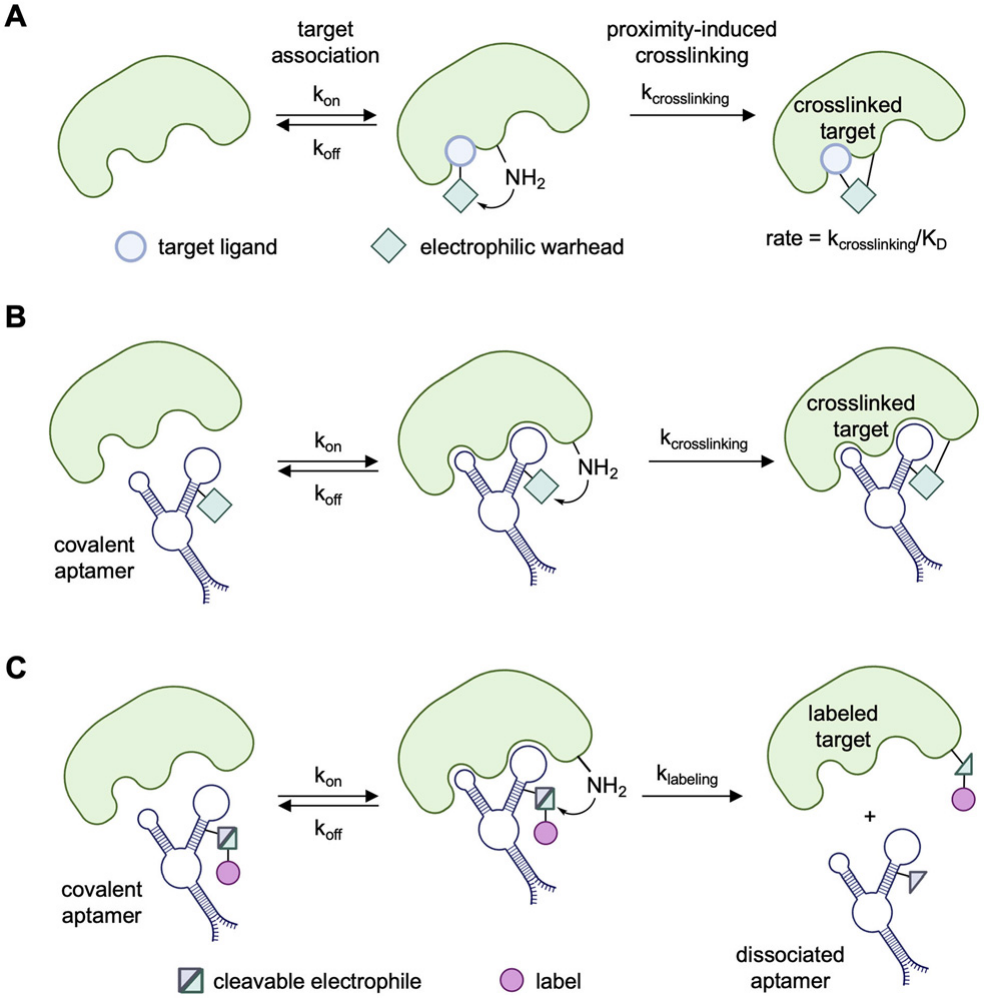
Small molecule- and aptamer-mediated covalent modification of proteins. Irreversible crosslinking of (A) a small molecule ligand or (B) an aptamer to its protein target *via* a proximity-induced reaction between an electrophile and a nucleophile. (C) Aptamers equipped with a cleavable electrophile transfer a label to a proximal nucleophilic residue on a target protein and are then free to dissociate.

The generation of covalent aptamers echoes that of covalent small molecule inhibitors, which are derived from known ligands and are similarly functionalized with electrophilic warheads capable of targeting nucleophilic amino acids, particularly serine, cysteine and lysine.^72^ Small molecule non-covalent inhibitors can associate with and dissociate from the protein target, as determined by *k*_on_ and *k*_off_ rates (Fig. 3A). Together, the on and off rates afford the dissociation constant *K*_D_. However, the covalency of crosslinking prevents the dissociation of the ligand from the small molecule-protein complex and thereby introduces the irreversible rate of *k*_crosslinking_. As such, the labeling reaction of covalent inhibitors is dependent upon both the affinity of the ligand and the reaction rate of the covalent inhibitor, where the second-order rate constant can be described as *k*_crosslinking_/*K*_D_. Therefore, a higher-affinity ligand (smaller *K*_D_ value) and/or a more reactive electrophile (higher *k*_crosslinking_ value) will result in a faster reaction rate.^73^ These small molecule ligands, referred to as targeted covalent inhibitors, show prolonged target engagement, conferring sustained inactivation, high specificity, and increased potency. The ensuing prolonged inhibition allows for dosage at lower concentrations, leading to less toxicity. There are more than 50 FDA-approved covalent drugs^74^ for diseases such as cancer,^75–80^ bacterial infection,^81^ viral infection,^82^ gastrointestinal disease,^83^ cardiovascular disease,^84^ and sickle-cell anemia.^85^

To translate this chemistry to aptamers, electrophilic warheads can either be site-selectively incorporated within the nucleic acid sequences or tethered to either the 5′ or 3′ termini. DNA and RNA do contain some slightly nucleophilic functional groups at the backbone (phosphates), the sugar moiety of RNA (2′-hydroxy of the ribose sugar), and at their nucleobases (for example the N7 position of guanine). However, these are much less nucleophilic than many amino acid sidechains and are not reactive enough to quench the incorporated electrophilic motif. The lack of inherent nucleophilicity in DNA allows for the installation of chemical warheads bearing cleavable electrophiles and payloads for transfer. Binding of the armed covalent aptamer to its protein of interest positions the electrophile into proximity with nearby nucleophilic amino acids, allowing for covalent bond formation between the two molecules through either crosslinking (Fig. 3B) or label transfer (Fig. 3C), depending on the chemistry of the electrophile. Multiple methods of warhead incorporation exist, including conjugation of the warhead to an amine-modified terminus^86^ and attachment to alkyne-modified phosphoramidites, such as the ethynyl-deoxyuridine (EdU) and octadiynyl-deoxyuridine (OdU) nucleobases.^87^

**Fig. 4:**
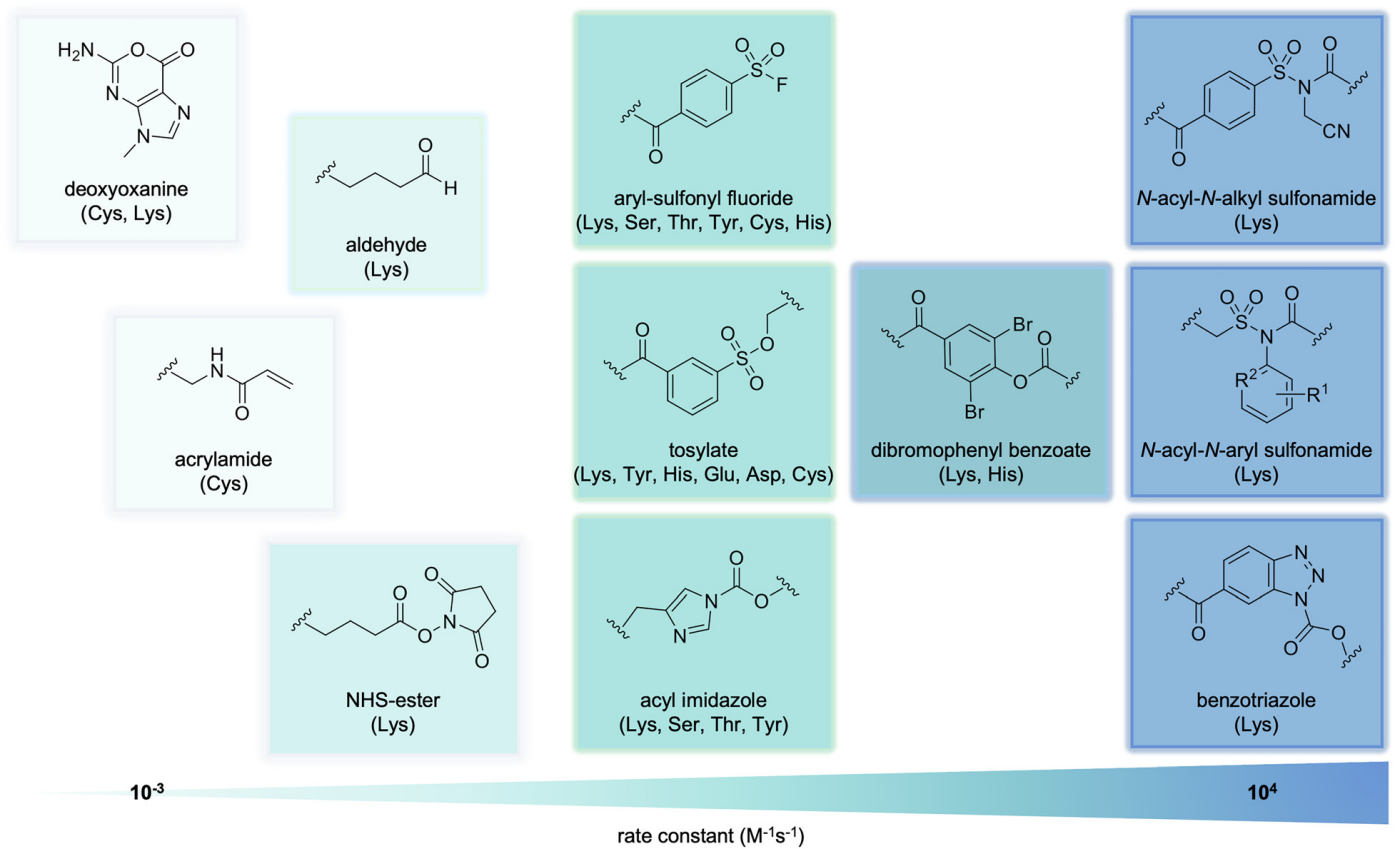
Electrophilic warheads that have been utilized for covalent protein modification arranged by the rate constant of label transfer. Amino acid side-chain specificity is listed in parentheses. R^1^ = Cl, Br, NO_2_, or F; R^2^ = CH or N.

The use of electrophilic warheads to arm covalent aptamers has varied widely by the reactivity and stability of the electrophile and by its selectivity for certain amino acid side chains. As previously mentioned, the first covalent aptamer developed contained arguably the simplest motif, an acrylamide (Fig. 4). Acrylamides specifically crosslink with cysteine sidechains, which are one of the least common amino acids in the proteome.^88,89^ The kinetics of acrylamides can be tuned through chemical modifications, though they maintain a rate around ∼100 M^−1^ s^−1^.^89^ Many small molecule covalent modifiers possess acrylamide motifs, and mutations of cysteines to the less nucleophilic serine often infers resistance of these proteins to modification. Crosslinking aptamers bearing aldehydes have also been developed, though they also have slow reaction rates, around 1 M^−1^ s^−1^. While aldehydes mostly target lysines, mass spec analysis has confirmed modification of other nucleophilic amino acid side chains, such as cysteine and histidine, and with N-terminal amino groups, though these interactions demonstrate instability and must be stabilized *via* borohydride reduction.^90,91^

The naturally occurring deoxyoxanine nucleobase (Fig. 4), which endogenously forms in cellular environments due to deamination of guanine, is capable of forming a covalent bond with an amino group.^92–94^ This can be a crosslink with a nearby DNA nucleobase or lysine sidechain on a protein surface. Lower reactivity has been observed with cysteine, arginine, and histidine residues.^95^ The electrophile, a cyclic *O*-acylisourea moiety, is remarkably stable toward hydrolysis. However, this also results in slow reactions, with rates as low as 10^−3^ M^−1^ s^−1^.^96^

As a result of its synthetic accessibility and high reactivity, the most popular electrophile for crosslinking aptamers is the *N*-hydroxysuccinimide (NHS) ester (Fig. 4). NHS esters can label lysine and N-terminal amines with high reaction rates, and to a much lesser extent serine, threonine, and tyrosine residues.^97,98^ Reactions of NHS esters are relatively slow, with a rate of about 1 M^−1^ s^−1^. While the amide product formed from the nucleophilic attack is quite stable, NHS esters themselves show limited stability under aqueous conditions (half-life of ∼4–5 h at pH 7), which further decreases at higher pH and temperature.^99^

Overcoming the limited aqueous stability of NHS esters, the aryl sulfonyl fluoride (ASF) moiety has been incorporated into covalent aptamer designs (Fig. 4). ASF electrophiles label nucleophilic residues on proteins through sulfur(vi)–fluoride exchange (SuFEx) chemistry, show quick reaction rates (∼10^6^ M^−1^ h^−1^),^100^ and good stability in aqueous conditions (*t*_1/2_ of hydrolysis of up to 25 h in rat plasma).^101^ However, the increased reactivity results in amino acid promiscuity, as the ASF warhead reacts not only with lysine, but also serine, threonine, tyrosine, cysteine, and histidine residues.^102^

While these electrophiles result in crosslinking of the aptamer to its protein of interest, ligand-directed (LD) chemistry, instead allows for traceless surface labeling of target proteins *via* cleavable electrophiles. In the context of small molecule ligands, this approach has been extensively developed by the Hamachi group,^103,104^ who studied a variety of electrophiles. These include tosylates, acyl-imidazoles, dibromophenyl benzoates, benzotriazoles, and *N*-acyl-*N*-alkyl-sulfonamides (NASA, Fig. 4). The reactivities of these electrophiles cover a range of reaction rates and specificities, such as labeling of lysine, threonine, tyrosine, histidine, and serine side chains. They have been used for the specific, covalent modification of dihydrofolate reductase, folate receptor, carbonic anhydrase II and XII, Bruton tyrosine kinase (BTK), and NADH-quinone oxidoreductase, among others.^105–110^

The NASA warhead has particularly desirable characteristics, such as excellent reactivity and selective labeling of lysine residues. While aqueous stability is limited, with a *t*_1/2_ of ∼8 h at pH 7, it is still slightly better than the previous generations of LD electrophiles. To improve the stability of the NASA electrophile, the Hamachi group developed a second-generation NASA electrophile, the *N*-acyl-*N*-aryl-sulfonamide (ArNASA).^108^ While maintaining lysine specificity and high reactivity rates, ArNASA displays a *t*_1/2_ of hydrolysis of >24 hours at pH 7, showing a surprising stability in aqueous conditions. This new generation of LD electrophiles has been used to modify BTK and heat shock protein 90.^108^

## 3. Aptamer-directed protein crosslinking with aldehyde and difluoromethylene-carboxyl groups

**Fig. 5:**
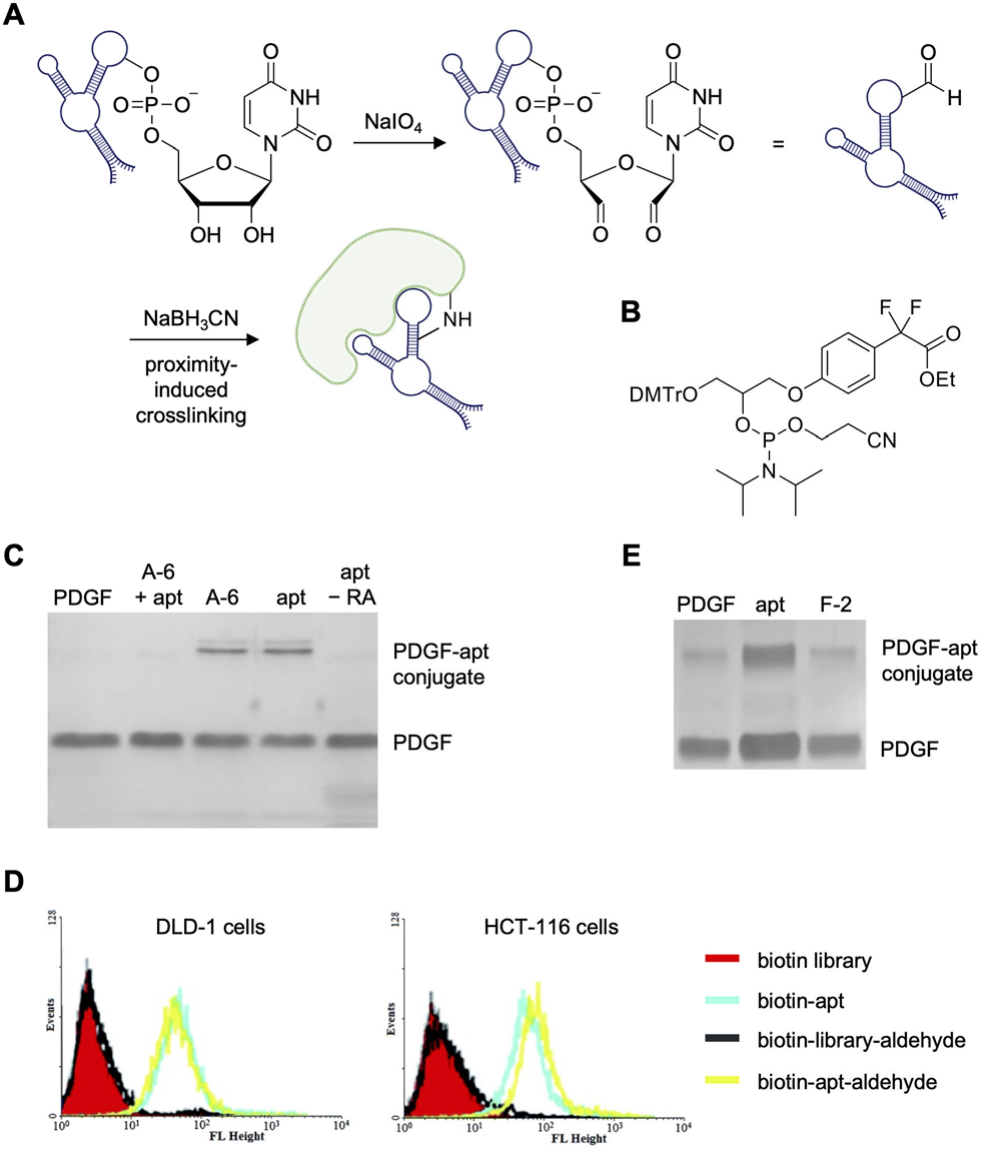
Aldehyde and difluoromethyl-carboxyl modified electrophiles in aptamer crosslinking. (A) Crosslinking of an aldehyde-modified aptamer with its protein target. (B) Structure of synthesized F-carboxyl phosphoramidite. Crosslinking of (C) aldehyde-modified PDGF aptamers and (D) *K*_D_ED2a (DLD-1 cells) and KCHA10a (HCT116 cells) aptamers modified with a 5′ biotin and a 3′ aldehyde specifically bound target cell lines as measured by flow cytometry using a streptavidin-phycoerythrin dye. (E) Crosslinking of PDGF with F-carboxy-modified PDGF aptamers. This figure was reproduced from ref. 111.

An early example of aptamer-mediated covalent modification was the Tan lab's reported protein–aptamer template for site-selective installation of DNA on protein surfaces.^111^ Target proteins were tagged with DNA aptamers *via* a one-step crosslinking method for cancer biomarker isolation and characterization. Two electrophiles were tested by installing either an aldehyde at the 3′ end through oxidation (Fig. 5A) or a α,α-*gem*-difluoromethyl carboxyl group (F-carboxyl; Fig. 5B) at the 5′ terminus of the aptamer and several linker lengths were explored.^111^ Thrombin and a series of RNA-CpG (cytosine–phosphate–guanine) thrombin-targeting aptamers were used as initial model systems. Here, recombinant protein and the aldehyde-modified aptamer were incubated in the presence of sodium cyanoborohydride (NaBH_3_CN) allowing for reductive amination of protein surface lysines to form irreversibly crosslinked aptamer–protein products. The methodology was also applied to platelet-derived growth factor-BB (PDGF-BB) and its aptamer. Here, moderate crosslinking (∼50%) to the target protein was observed (Fig. 5C); however, a control aptamer (A-6) armed with the same aldehyde electrophile showed the same level of crosslinking. While this nonspecific interaction could be mitigated through incubation with unmodified PDGF aptamer, the Tan lab transitioned to alternative crosslinking moieties.

Prior to changing moieties, however, the Tan lab showcased covalent crosslinking in a cellular context, aptamers that bind the colorectal cancer cell lines DLD-1 and HCT116, but whose protein targets are unknown, were functionalized with terminal aldehydes.^112^ Here, 5′ termini of aptamers specific for each cell line were appended with a biotin while the 3′ termini were oxidized to leave the reactive aldehyde group. Streptavidin modified with Cy5 dyes was added to cells and the binding was detected *via* flow cytometry. As a nonspecific control, the experiment was repeated with a randomized library of aldehyde-modified aptamers, which showed no binding to the colorectal cancer cells (Fig. 5E).

In an attempt to increase crosslinking efficiency, the Tan lab synthesized an F-carboxyl modified phosphoramidite and generated aptamers with this reactive group at the 5′ end. About 50% crosslinking between the aptamer (F-1) and PDGF (Fig. 5D) was observed and, unlike in experiments with the aldehyde warhead, non-specific crosslinking of the control covalent aptamer (F-2) did not occur. Though the cell labeling appears to be quantitative (Fig. 5E), only partial crosslinking was observed with the aldehyde and F-carboxyl electrophiles *in vitro* and left room for improvement with a more reactive motif. Further directions could include identifying the unknown cell surface binding protein in these cell lines *via* covalent crosslinking and subsequent pull down. Additionally, mass spec analysis of the crosslinked conjugate was not reported, which could give further insight into the binding mechanisms of these aptamers.

## 4. Aptamer-directed protein crosslinking with *N*-hydroxysuccinimide (NHS) esters

*N*-Hydroxysuccinimide-esters (NHS esters) have been employed widely in chemical biology for bioconjugation to nucleophilic amino acid residues. These electrophilic esters react readily with lysine residues, and to a lesser extent with serine, threonine, and tyrosine residues.^97,98^ Not surprisingly, NHS esters were installed on aptamers for crosslinking to target proteins.

**Fig. 6:**
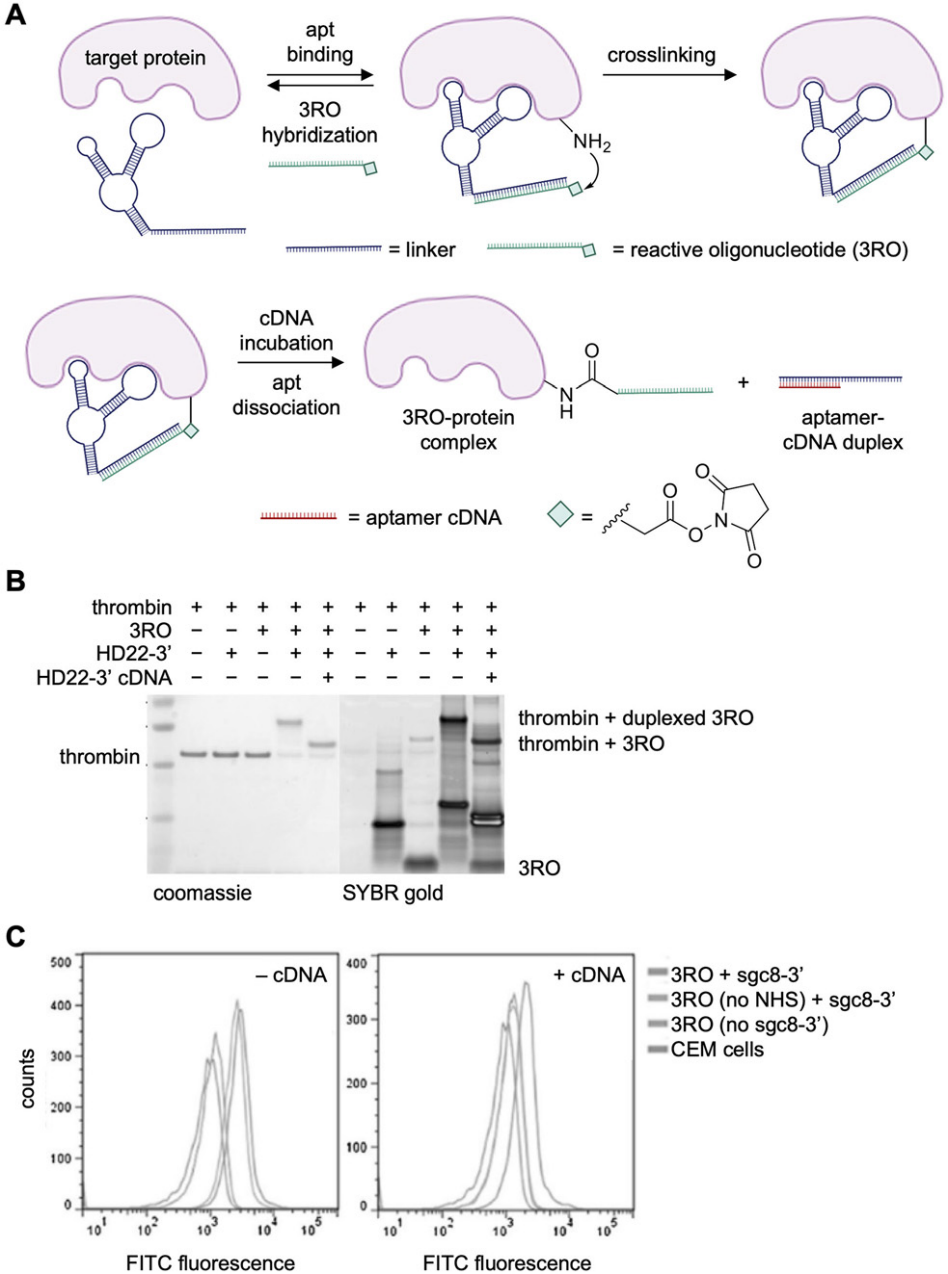
Reactive oligonucleotide crosslinking *via* an NHS ester. (A) An aptamer containing a linker sequence binds to a complementary reactive oligonucleotide modified with an NHS ester. Upon target binding, nucleophilic attack onto the ester crosslinks the aptamer to the protein. (B) SDS-PAGE analysis of DNA–protein complex formation with thrombin and its aptamer (HD22), the 3′-modified RO, and the blocking cDNA oligonucleotide. (C) Flow cytometry results of aptamer-mediated conjugation of PTK7 on CEM cells. Parts of this figure were reproduced from ref. 86 with permission from John Wiley and Sons, copyright 2017.

As a first example, the Tan lab utilized NHS ester chemistry to generate a covalent thrombin-binding aptamer (TBA). The design utilized a 23-nucleotide linker that was recognized by a complementary “reacting oligonucleotide” (RO) carrying a NHS ester on its 3′ terminus (Fig. 6A).^86^ Utilizing DNA duplex formation for presentation of the NHS ester, as opposed to chemically installing in on the aptamer itself, allows for generalization of a single NHS-modified RO to multiple aptamers. Upon hybridization of the RO strand, the electrophile could crosslink to a proximal lysine residue on the target protein (lanes 4 & 9, Fig. 6B). Crosslinking of the RO did not occur without the proximity afforded by the aptamer (lanes 3 & 8, Fig. 6B). Addition of a full-length complement oligonucleotide released the aptamer from the complex, leaving the protein-RO conjugate (Fig. 6B, lanes 5 & 10). A significant percentage of the protein was crosslinked (85%) after one hour incubation with 2 equivalents of aptamer and RO. RO crosslinking was specific for thrombin in the presence of the off-target protein bovine serum albumin (BSA). Mass spectrometry revealed the modification of thrombin at K57 and K288 residues with both the 3′- and 5′-modified ROs.

Modification of CCRF-CEM cell surfaces was explored next by lengthening by installing the linker strand on the well-established sgc8 aptamer targeting the cell surface cancer antigen PTK7.^113^ Using an RO bearing a fluorescein dye and the sgc8c aptamer, specific covalent delivery of the RO strand to PTK-7 expressing cells was observed along with negligible labeling of PTK7-negative Ramos cells (Fig. 6C).^113^ Taken together, this showed efficient crosslinking to thrombin and cell surface proteins, engaging a ternary complex between the target protein, the aptamer, and a hybridized reactive DNA strand. Further studies may be helpful in determining potential site-selectivity of this approach of crosslinking to nucleophilic amino acid sidechains.

**Fig. 7:**
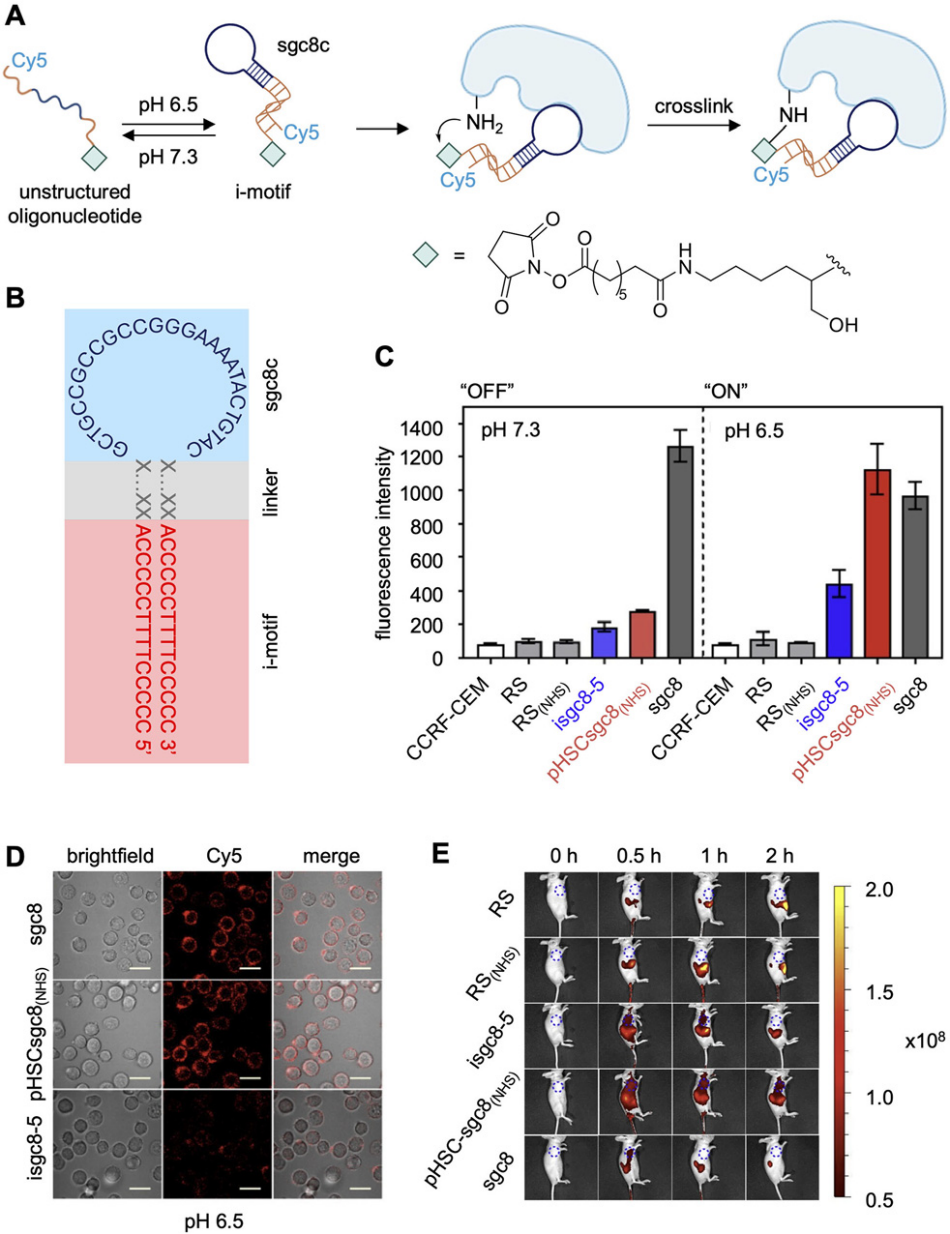
pH responsive aptamer crosslinking. (A) The aptamer “ON” folded conformation (pH = 6.5) allows for target binding and covalent lysine modification by an NHS ester. (B) Sequence of the isgc8-5 aptamer. (C) OFF (pH 7.4) *vs*. ON (pH 6.5) aptamer-meditated labeling of PTK7 cell surface protein, quantified by flow cytometry. (D) Micrographs of cells labeled by fluorescently tagged aptamers at pH 6.5. (E) Increased accumulation of pHSCsgc8_(NHS)_ at CCRF-CEM tumor xenografts in mice after 24 h. Parts of this figure were reproduced from ref. 114 and 116 with permission from American Chemical Society, copyright 2022 and 2018.

The specificity of aptamers can be further enhanced by conditional control in a temporal and spatial fashion. For example, tumor microenvironments maintain uniquely acidic pH levels compared to the rest of an organism. The Tan group developed pH-responsive NHS ester-modified aptamers to activate crosslinking solely to tumor cells expressing a desired biomarker.^114^ The aforementioned PTK7 aptamer, sgc8c, was fused to a split, intercalated-DNA motif (i-motif) which takes on a folded structure only in the acidic tumor microenvironment (pH = 6.5) due to the stabilization of intercalated, cytosine-cytosine base pairs (Fig. 7A).^115^ Since sgc8c binding was maintained with only the loop region of the folded aptamer (Fig. 7B), the stem portion was replaced with the cytosine-rich i-motif sequence.^116^ At physiological conditions, the i-motif is unstructured, preventing proper sgc8c folding and therefore PTK7 recognition.^116^ After exposure to acidic pH, the pH-responsive DNA probe folds into its native structure allowing for crucial surface binding interactions to occur between aptamer and the target protein (Fig. 7A). Then, the NHS ester installed at the 3′ terminus of the aptamer reacts with a proximal lysine residue on the protein surface.^86^ Flow cytometry analysis showed that the aptamer bound the target cells at both low pH (6.5) and at normal biological pH (7.3). In contrast, the pH-responsive covalent aptamer, pHSCsgc8_(NHS)_, only bound the PTK7-positive CCRF-CEM cells at low pH (Fig. 7C). A lower dissociation constant was observed with the covalent aptamer (*K*_d_ = 13.2 ± 1.5 nM) compared to the non-covalent aptamer, isgc8-5 (*K*_d_ = 66.4 ± 5.4 nM), assumed to be due to an off-rate of zero for aptamers that are covalently engaged with PTK7. These results were confirmed *via* confocal imaging, as the covalent aptamer showed more intense labeling of PTK7 positive cells, compared to its non-covalent, pH-dependent counterpart (Fig. 7D). However, labeling was comparable with the non-modified sgc8 aptamer at this lower pH. When injected into mice, pHSCsgc8_(NHS)_ exhibited increased accumulation at tumor xenografts, compared to sgc8c without a crosslinking electrophile (Fig. 7E), likely due to increased stability and residence time after covalent bond formation.^114^ Mass spectrometry studies revealing the extent of modification and sites modified on the PTK7 surface would provide additional important information.

**Fig. 8:**
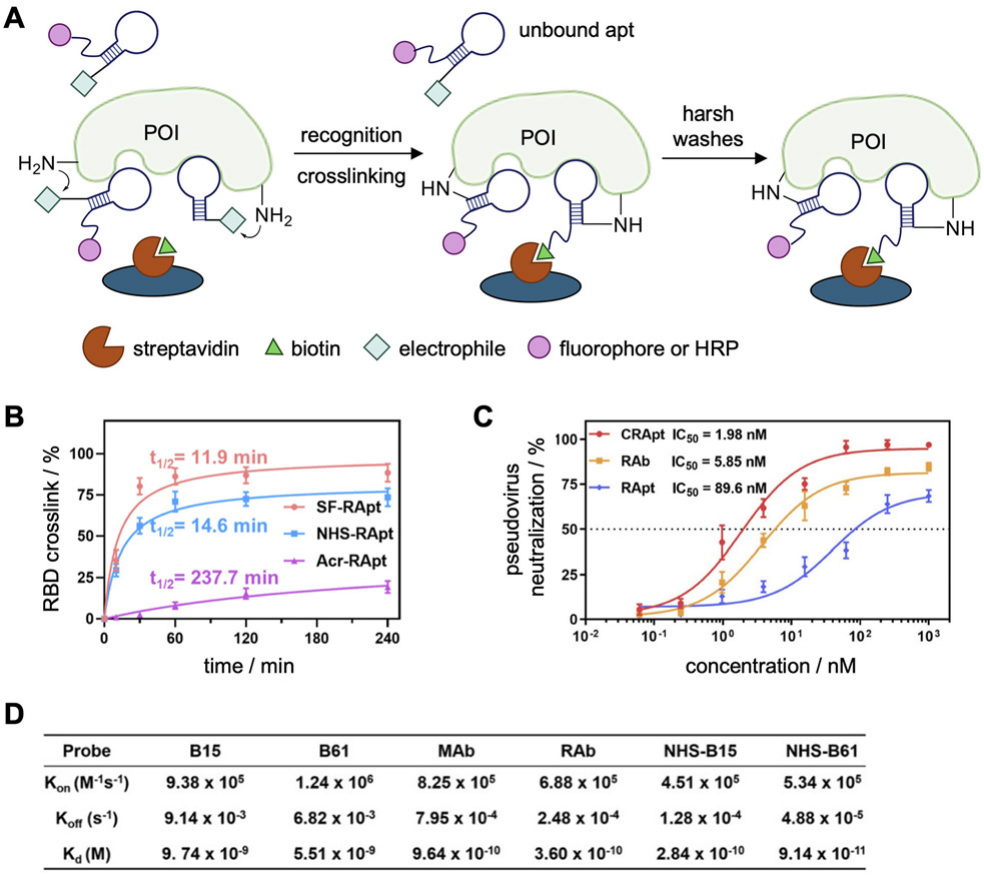
Crosslinking aptamers targeting SARS-CoV-2 proteins. (A) Schematic of a covalent ELISA with increased sensitivity due to harsh formamide and tween-20 washes to remove unbound aptamers. (B) Labeling kinetics of the three covalent aptamers. (C) Evaluation of pseudovirus neutralization using either NHS-CRapt, RAb, and RApt. (D) *K*_d_ measurements of different NP probes by SPR. Parts of this figure were reproduced from ref. 127.

In light of the recent global pandemic several aptamers targeting components of the SARS-CoV-2 virus have been reported, including the full Spike protein (S1),^117–121^ receptor binding domain (RBD),^39,40,118,120,122^ N-terminal domain (NTD),^123^ the trimeric S protein,^117,118,124,125^ and nucleocapsid protein (NP).^126^ Aptamers targeting the nucleocapsid protein (NP) and the receptor binding domain (RBD) of the spike protein of SARS-CoV-2 were functionalized with electrophiles.^127^ NP is the preferred target for early detection of SARS-CoV-2 as it is the most conserved and abundant structural protein. Tangentially, ELISA assays are the gold standard for high-throughput detection of viral infection, and the Tan lab hoped to increase the sensitivity of these assays through covalent aptamer–protein complex formation to allow for harsher washes of non-specific probe binding (Fig. 8A).^127^ Here, the presence of a protein of interest (POI) is detected *via* immobilization of a “capture probe,” typically an antibody or aptamer. Then, the sample is added, capturing the POI, which can be read out through binding of a “detection probe,” typically a labeled antibody or aptamer producing fluorescence or chemiluminescence, and was used to characterize the binding of the aptamers to their target proteins. For comparison to covalent aptamers (NHS-Apt), a monoclonal antibody (MAb), a commercially available rabbit antibody (rAb), and a non-covalent aptamer were used. The harsher wash of a sodium citrate buffer containing 10% formamide (10%) and tween-20 (1%) significantly reduced the limit of detection of the viral probes and was utilized to display the increased sensitivity of the covalent functionalization.

To increase the sensitivity of these diagnostic assays, an aryl sulfonyl fluoride (ASF), an NHS ester, or an acrylamide (Acr) (see Fig. 4 for structures) was installed at the 5′-terminus of the aptamers.^122,126^ Examination of the crosslinking efficiency, specificity, and kinetics of all three warhead-functionalized NP aptamers revealed that the NHS modification is optimal for covalent bond formation to NP, with high efficacy (81%) and fast kinetics (*t*_1/2_ = 22.8 min, Fig. 8B); however, an excess of 6 equivalents of aptamer was used to achieve these results. While the SF-NP aptamer showed even higher crosslinking efficacy (95%) and faster kinetics (*t*_1/2_ = 12.7 min), it produced undesired crosslinks with the off-target protein HSA (human serum albumin).^128^ The authors hypothesized that off-target crosslinking to HSA was due the electrophile's higher reactivity, increased stability (*t*_1/2_ ∼25 h in biological conditions^101^) and the use of excess off-target protein (10 : 1 HSA to NP). Meanwhile, the acrylamide warhead displayed slow kinetics and did not efficiently label either target. This poor reactivity is due to a combination of its lower electrophilicity compared to NHS and ASF warheads and the lack of cysteine residues on the surface of NP (0) and RBD (4).

While the covalent NP aptamers demonstrated increased detection sensitivity through a covalent ELISA assay, the covalent RBD aptamers were utilized in a neutralization assay to showcase the therapeutic potential of this approach. Once the aptamers are bound to RBD, the virus is unable to bind the ACE2 receptor, therefore preventing viral infection of a host cell. The neutralization ability was determined through infection of ACE2-expressing HEK293T cells with luciferase-tagged pseudovirus. The CRApt (IC_50_ = 2 nM) displayed an increased ability to neutralize infection compared to the noncovalent aptamer (IC_50_ = 80 nM), and comparable neutralization to the antibody (IC_50_ = 6 nM, Fig. 8C).

To validate that the increased sensitivity of detection and ability of virus neutralization were a result of tighter dissociation constants afforded by the covalency, ELISA analyses were performed analyzing the binding constants for the NHS-B15 and NHS-B61 aptamers compared to the non-covalent B15 and B61 aptamer complexes. While significant dissociation of the non-covalent B15 and B61 aptamer complexes was detected, the *k*_off_ rates for the covalent aptamers were 1–2 full orders of magnitude slower (Fig. 8D). As there was only a negligible difference in the *k*_on_ rates for the covalent *vs.* non-covalent aptamers, an improved *K*_D_ value was obtained for the covalent aptamers. Additionally, the *K*_D_ of the NHS-aptamers was comparable or even less than that of the corresponding antibodies. It is important to note that the *k*_off_ values were not zero for the covalent complexes as the crosslinking efficiency was not 100%. Lastly, mass spectrometry analyses revealed that K417 of RBD, which participates in a key interaction to promote viral infection, was the site of crosslinking. These experiments highlight promise towards the use of covalent aptamers as diagnostic and therapeutic tools for viral infections.

**Fig. 9:**
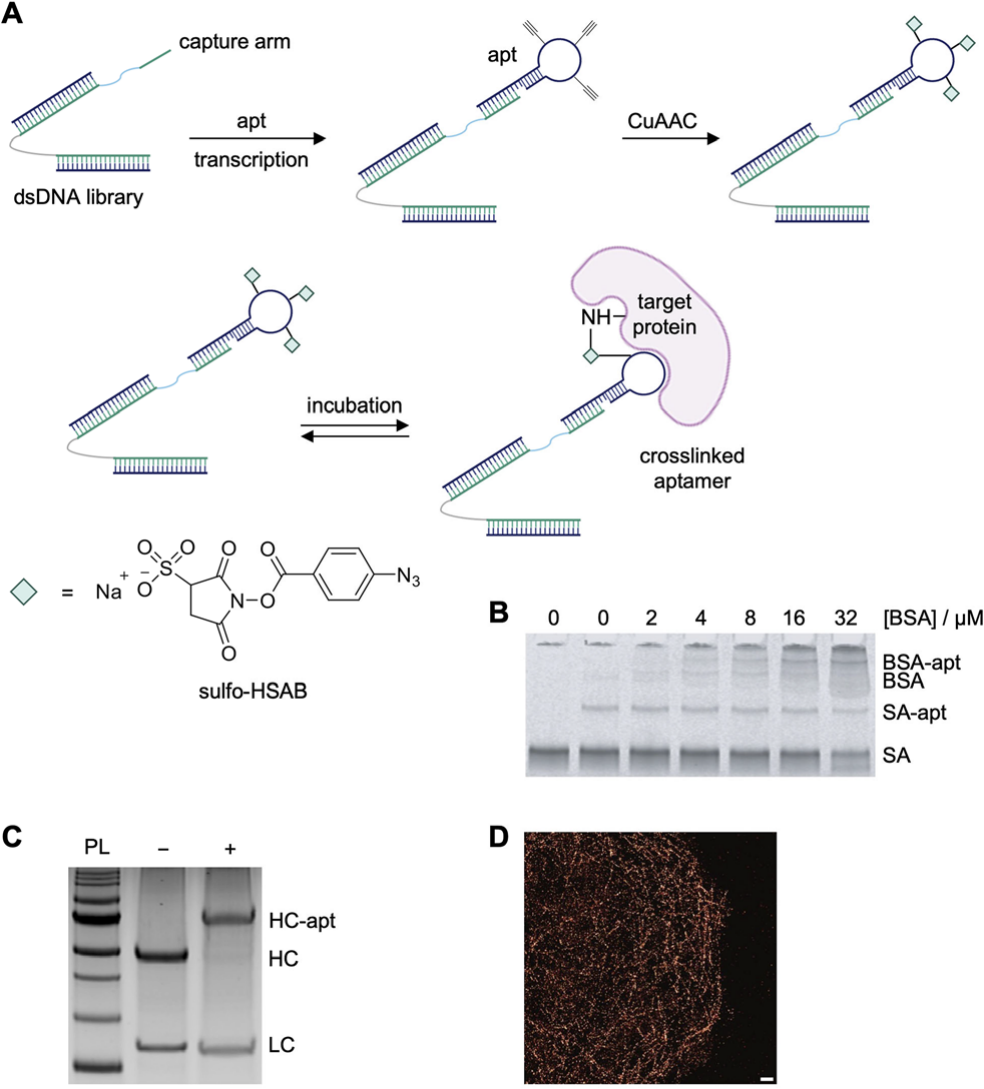
Selection of aptamers for streptavidin and monoclonal antibodies using SELMA. (A) RNA-SELMA library generation, functionalization, and crosslinking scheme. (B) SDS-PAGE analysis of crosslinking specificity of the selected streptavidin aptamer with increasing concentrations of BSA. (C) Mouse IgG antibody was incubated with selected crosslinking aptamer and analyzed *via* reducing SDS-PAGE. (D) DNA-paint image of microtubules. Scale bar: 5 μm. Parts of this figure were reproduced from ref. 129 and 131.

The MacPherson lab developed an RNA-SELMA (selection with modified aptamers) strategy that enables the *de novo* selection of covalent RNA aptamers by incorporating *N*-hydroxysuccinimide (NHS) esters into an RNA library (Fig. 9A).^129^ This approach adapts elements of click-SELEX, where ethynyl-modified nucleotides (such as 5-ethynyluridine triphosphate) are incorporated in place of all uridine residues during *in vitro* transcription, allowing for the subsequent attachment of electrophiles *via* copper-catalyzed azide–alkyne cycloaddition.^130^ The resulting RNA library, which is physically linked to its encoding DNA through a “capture arm” sequence, was thus equipped with multiple NHS-ester groups per oligonucleotide, creating “barbed” aptamers capable of covalently reacting with protein targets. Following modification of the alkyne-incorporated RNA with sulfo-HSAB, the NHS-ester-functionalized RNA library was incubated with the target protein (streptavidin), leading to covalent bond formation between the aptamer and accessible lysine residues on the protein. Covalently crosslinked RNA–protein complexes were selectively retained and the corresponding, attached DNA templates were PCR-amplified to regenerate the library for subsequent rounds of selection. To enhance specificity, six of the sixteen selection rounds included negative selection steps using bovine serum albumin (BSA) as an off-target competitor, with both denatured and native BSA employed at increasing stringency in later rounds. Despite the covalent nature of the selection, a relatively high number of rounds was required to achieve enrichment, likely reflecting the challenge of isolating highly specific covalent binders from a diverse and reactive library.

No binding of streptavidin by the selected aptamer with canonical uridines as opposed to the ethynyluridine bases used during selection was detected in gel shift assays, indicating that the electrophile was necessary for the interaction with streptavidin. Additionally, incomplete crosslinking to streptavidin (35%) and off-target crosslinking to BSA was observed (Fig. 9B). It is interesting that the off-target affinity remained despite negative selection rounds in the SELMA process. Nevertheless, covalent DNA aptamers were selected for the Fc domain of mouse monoclonal antibodies (mAbs) using this approach. The mAb-targeting covalent aptamers showed near complete crosslinking to the heavy chain of the antibody (95% modified, Fig. 9C). MS analysis of crosslinked residues was attempted but was unsuccessful and no information on target residue specificity was obtained. The aptamers were used in a DNA-PAINT imaging application, where a fluorophore-modified cDNA bound the antibody–aptamer conjugate (Fig. 9D), showcasing a unique application of covalent aptamers.^131^

Despite the development of this particular streptavidin-targeting RNA aptamer, the majority of aptamers functionalized with electrophilic warheads have been DNA aptamers, likely because RNA is more susceptible to hydrolysis and degradation.^132^ Moreover, RNAs carry a nucleophilic 2′ OH group, which has been taken advantage of in strategies like selective 2′-hydroxyl acylation analyzed by primer extension (SHAPE) reagents. These are based on activated esters, such as isatoic anhydride or benzoyl cyanide derivatives, and are utilized to probe RNA structure and dynamics through acylation of the 2′ OH functional group.^133–136^ Thus, there is limited literature precedence for installing an electrophilic moiety onto an RNA molecule for subsequent covalent labeling or crosslinking to proteins. In one such instance, a tRNA with a 2′ azido modification at the 3′ terminus was synthesized and subsequently reduced and conjugated to diethylsquarate, an electrophile that can crosslink to proximal amines on an tRNA aminoacyltransferase.^137^ Together with another report of an RNA ligand equipped with a NASA electrophile for transfer of a hydrophobic tag to a target protein, this suggests that the reactivities of amine-reactive electrophiles and ribose 2′-hydroxyl groups are compatible.^138^ Regardless, the authors of the RNA-SELMA report themselves note the rarity and difficulty of generating base-modified RNA aptamers compared to DNA aptamers, and the need for careful handling and storage of NHS-ester-modified RNA due to its lability. In subsequent work, the same group focused on DNA aptamers for covalent crosslinking.

## 5. Aptamer-directed protein crosslinking with aryl sulfonyl fluoride (ASF)

Although NHS esters have a robust precedence for labeling of biomolecules, their susceptibility to hydrolysis in aqueous conditions^99^ remains a concern for translation *in vivo* and therefore other warheads such as arylsulfonyl fluorides (ASFs) were investigated to overcome these limitations. As previously discussed, ASF warheads possess favorable resistance to hydrolysis (*t*_1/2_ of 25 h in physiological conditions), with their reactivity being tunable depending on the substituents on the aryl ring. However, with a labeling rate constant of up to 1902 M^−1^ h^−1^, the ratio of labeling/hydrolysis can be as high as 5000-fold, suggesting ideal kinetics for aptamer-mediated crosslinking.^100,101^ Comparison of ASF warheads appended with different electron withdrawing groups showcased tunable rates and residue specificity, though in all cases the reaction rate was slower for lysine modification than tyrosine modification. Nevertheless, this suggested the ability to rationally design ASF warheads for the specific environment of a protein's binding pocket.^101^ Additionally, while NHS esters show specificity towards lysine residues, ASF warheads react with most nucleophilic residues in addition to lysines, including serine, threonine, tyrosine, cysteine, and histidine.^102^ This presents a higher probability for an appropriately reactive residue being present proximal to the aptamer binding site. Taken together, hydrolytic stability and extended reactivity toward a range of nucleophiles make ASF a viable candidate for aptamer–protein crosslinking.

**Fig. 10:**
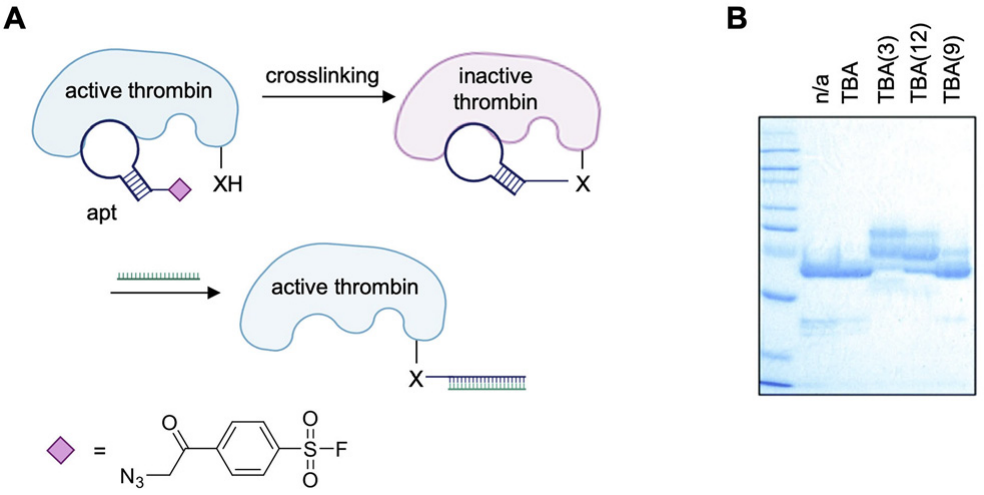
(A) Scheme of covalent crosslinking of aptamer to thrombin (Nu = Y88 and H91) and subsequent reversibility of protein inhibition through hybridization to a complementary strand. Structure of the ASF warhead. (B) SDS-PAGE analysis of covalent aptamer–protein conjugates. Part of this figure was reproduced from ref. 140 with permission from Royal Society of Chemistry, copyright 2021.

The first aptamer equipped with an ASF warhead was the well-established thrombin binding aptamer (TBA; Fig. 10A).^139,140^ The electrophile was installed onto the DNA sequence through incorporation of an alkyne-modified nucleobase during synthesis and subsequent CuAAC conjugation to an azide-modified electrophile.^140^ Unlike the reports previously discussed where the electrophile was tethered to either the 5′ or 3′-terminus of the aptamer, the ASF warheads were site-specifically installed at position 3, 9, and 12 of the aptamer. These sites were chosen for their positioning either facing towards (positions 3 and 12) or away (position 9) from the TBA/thrombin interface based on the crystal structure.^141^ Though all positions were capable of crosslinking thrombin, TBA(3) exhibiting the greatest efficiency at approximately 90% crosslinked thrombin (Fig. 10B).^140^

Only minimal crosslinking was observed with TBA(9), facing away from the TBA/thrombin interface, confirming the covalent bond formation is resultant of correct positioning of the electrophile. Mass spec sequencing of thrombin crosslinked to TBA(3) revealed that the amino acids Y88 and H91 were modified, which was rationalized through the reported crystal structure of TBA(3) and thrombin in complex, showing the electrophile positioned proximally to these nucleophilic residues. It would have been interesting to see if mass spec sequencing of thrombin crosslinked to TBA(9) resulted in modification of different residues to confirm the hypothesis of multiple aptamer binding sites.

**Fig. 11:**
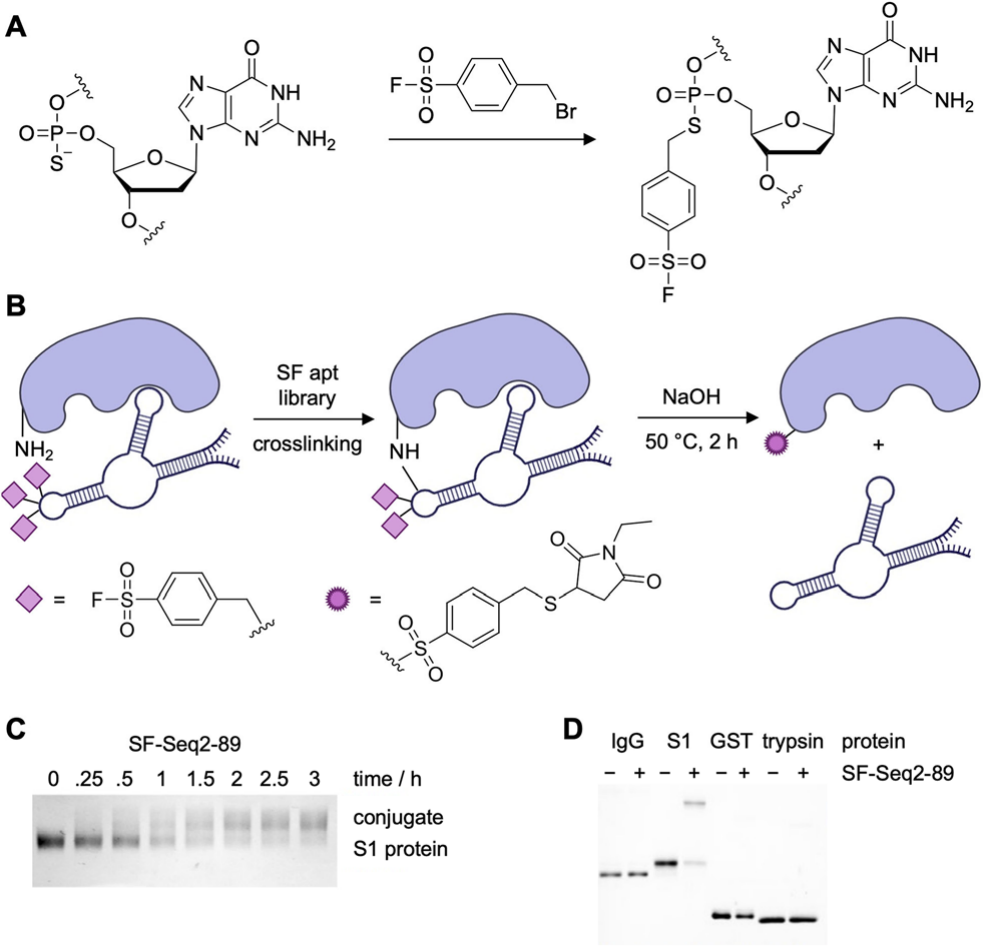
(A) Reactive ASF warheads installation into the backbone of a site-specifically PS modified DNA library. (B) Schematic depicting SF-aptamer crosslinking proteins, followed by cleavage, reduction, and alkylation *via N*-ethylmaleimide (NEM) for mass spectrometry analysis. (C) SDS-PAGE analysis of time dependency study of the reaction between S1 and SF-Seq2-89. (D) SDS-PAGE analysis of specific SF-Seq2-89 crosslinking to the RBD of S-protein but no other relevant off-target proteins. Parts of this figure were adapted from ref. 57 from Springer Nature, copyright 2023.

ASF warheads were also employed in the generation of covalent aptamers that targeted the SARS-CoV-2 spike protein (S-protein) *via* an *in vitro* selection strategy (Fig. 11A).^57^ After library amplification, using dATP-α-S for specific PS installation, the ASF warhead was introduced through S_N_2 chemistry to generate crosslinking aptamers (Fig. 11B).^142^ Through the covalent *in vitro* selection strategy, two aptamers were selected targeting the S-protein. This approach to covalent SELEX was only slightly different then MacPherson's method for RNA-SELMA. While it included the rational placement of electrophiles, the number of selection rounds was similar (13 and 16 total, including 2 and 6 negative rounds). Qin *et al.* eluted their bound sequences through a denaturing urea PAGE and isolated the bands corresponding to the protein–aptamer complex. The gel slices were treated with stringent conditions (NaOH at 50 °C for 2 h) to remove the ASF modifications and release native DNA, a process more involved than dehybridization of the aptamer-encoding oligo in the case of RNA-SELMA.

Expanding on their covalent DNA SELEX approach, the Xiang group selected two aptamers for the RBD of SARS-CoV-2. While the binding constants of the covalent aptamers were not reported, the analogs without electrophiles installed displayed *K*_D_s as low as 2.05 nM, comparable to other aptamers selected for S-protein or receptor binding domain (RBD) of SARS-CoV-2.^40,121^ While it would have been compelling to compare the dissociation constants of both the unmodified and the ASF-modified aptamers, quantitative crosslinking of S-protein was observed with 6 equivalents of the aptamer SF-Seq2 within 3 hours (Fig. 11C). Specificity of SF-Seq2 was confirmed by incubating the aptamer with various off-target proteins, including IgG1 Fc, GST, and trypsin (Fig. 11D). It is interesting that there is more specificity with these aptamers compared to the streptavidin aptamers selected in MacPherson's RNA-SELMA approach, even though fewer negative selection rounds were employed.^129^

Mass spec sequencing revealed that the aptamer crosslinked at positions Y421 and K458, which are conserved residues across the different SARS-CoV-2 variant strains. These residues are closely situated in the protein structure, indicating that the aptamer binds at a proximal site. Though it is not reported how many warheads are engaged in covalent bond formation, the authors did note that reducing the number of electrophile modifications significantly reduced the ability of the aptamer to crosslink RBD and to inhibit the RBD–ACE2 interaction. Peptide mapping data showed that several different residues on the RBD (including K378, R408, Y422, Y424, Y453, and K458) are modified by the aptamer; however further mass spectrometry studies are needed to elucidate the relative crosslinking efficiency at each lysine residue. Overall, the crosslinking aptamers were capable of blocking the interaction between S-protein and the ACE2 receptor expressed on cells, and neutralized SARS-CoV-2 virus with 100-fold more potency (IC_50_ = 0.93 nM) than their non-covalent counterparts (IC_50_ = 110 nM).

**Fig. 12:**
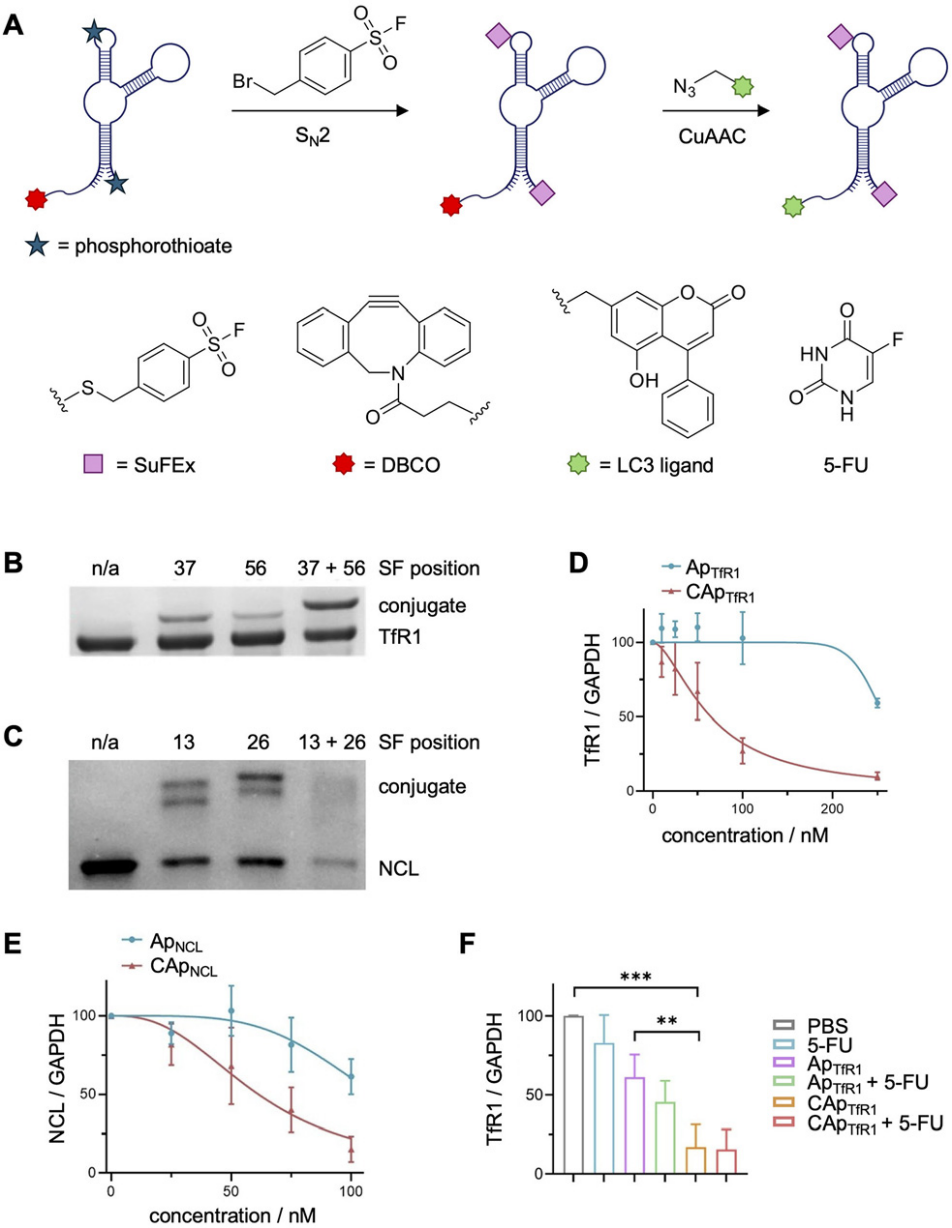
Covalent aptamer mediated autophagic degradation of membrane proteins. (A) Scheme depicting the synthesis of LC3-recruiting covalent aptamer chimeras. SDS-PAGE analysis of SuFEx-induced crosslinking to recombinant (B) TfR1 and (C) NCL. (D) TfR1 and (E) NCL degradation in HeLa cells emphasizes the necessity for covalent engagement. (F) TfR1 degradation was also observed in a mouse xenograft model. Parts of this figure were adapted from ref. 143 with permissions from John Wiley and Sons, copyright 2025.

The modification of PS backbones was also employed in the generation of ASF-modified covalent aptamers capable of initializing the autophagic degradation of proteins present on cell membranes (Fig. 12A).^143^ Here, covalent aptamers targeting transferrin receptor 1 (TfR1) and nucleolin (NCL) were generated through the incorporation of multiple PS modifications at defined backbone sites and were equipped with ASF warheads through the same chemistry. Aptamer crosslinking was assessed with recombinant TfR1 protein (Fig. 12B), comparing the efficiencies of aptamers with one or two warheads. Though the crosslinking efficiency significantly increased with the double modification, only a 42% crosslinking efficiency was observed when using an excess of 10 equivalents of the aptamer. In contrast, the nucleolin aptamer containing two warheads showed an excellent 95% crosslinking efficiency (Fig. 12C). Unfortunately, mass spec analysis of crosslinked residues was not performed but might reveal mechanistic insight as to whether the crosslinking efficiency increases due to targeting of different nucleophilic residues on the protein, as suggested by molecular modeling studies.

However, despite observing nearly complete target crosslinking with either aptamer, both covalent aptamers were conjugated with LC3 (microtubule-associated protein 1 light chain 3) ligand (Fig. 12A). Recruitment of LC3 to a protein of interest should initiate autophagic degradation as the membrane proteins are internalized through caveolin-mediated endocytosis. LC3 is hypothesized to interact with cargo receptors to recruit the marked proteins to the autophagosome, resulting in degradation.^144,145^ Here, degradation of 88% of TfR1 and 76% of NCL was observed in cell culture (12 h incubation, Fig. 12D and E), and a significant reduction of TfR1 levels was seen in a mouse xenograft model (Fig. 12F). Interestingly, HeLa cells treated with the covalent TfR1 aptamer had an increase in the population of S phase cells, indicating that the reduction in TfR1 levels promoted cell cycle arrest. These results inspired a combined aptamer treatment with 5-fluorouracil (5-FU), which displays cell-cycle dependent cytotoxic effects for a synergistic treatment. The co-treatment led to a significant increase in cytotoxicity compared to either treatment independently and concomitant suppression of tumor growth in mice. These studies represent the second example of covalent aptamers showing efficacy *in vivo*, highlighting the therapeutic promise of this approach.

## 6. Aptamer-directed protein crosslinking with a deoxyoxanosine electrophile

**Fig. 13:**
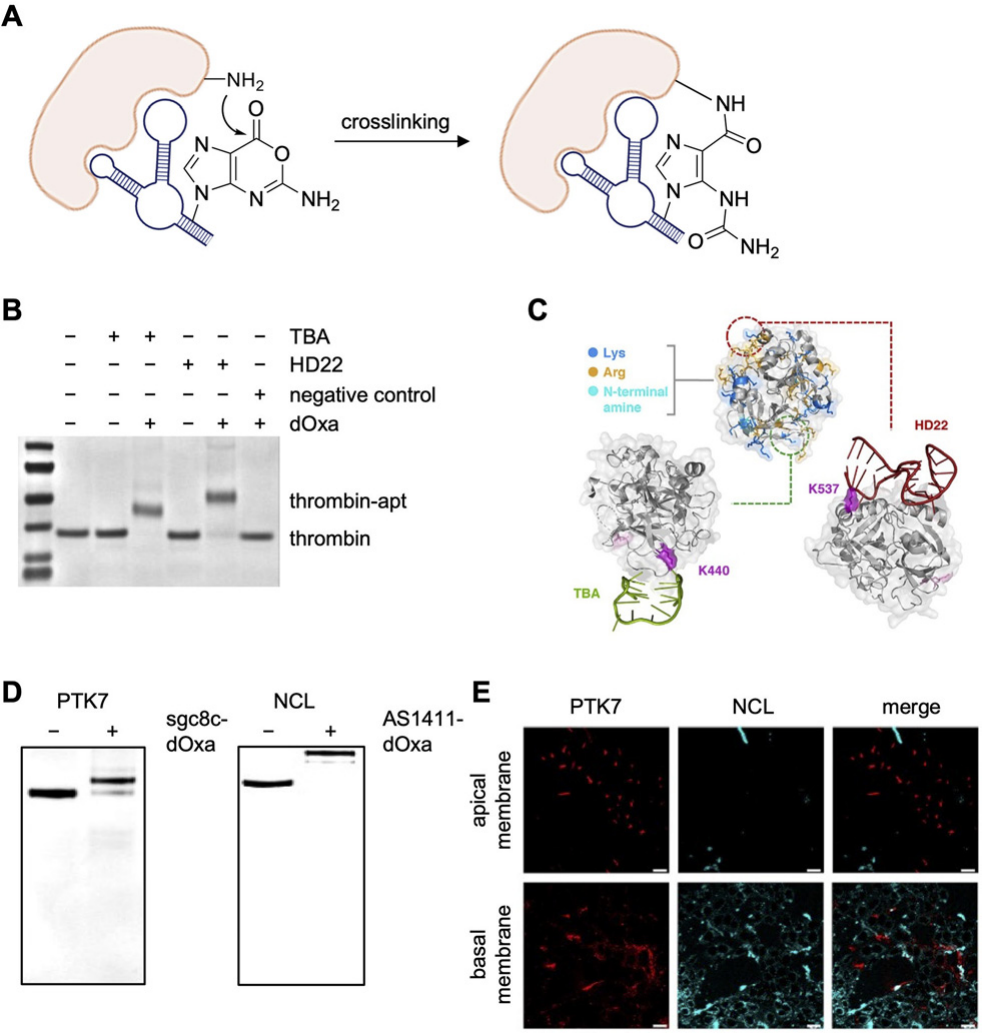
Protein crosslinking with deoxyoxanosine (dOxa)-modified aptamers. (A) Mechanism of amine–oxanine crosslinking. (B) SDS-PAGE analysis of TBA-dOxa and HD22-dOxa crosslinking to thrombin. (C) Mapping of modified lysine residues onto protein–aptamer structures. (D) SDS-PAGE analysis of sgc8c-dOxa and AS1411-dOxa crosslinking to PTK7 and nucleolin, respectively. (E) Imaging of PTK7 and nucleolin (NCL) with sgc8c-dOxa, AS1411-dOxa, and their respective fluorophore-tagged cDNA (MCF7 cells). Scale bar is 20 μm. Parts of this figure were adapted from ref. 95 with permission from the American Chemical Society, copyright 2025.

All electrophiles discussed thus far suffer, to varying degrees, from some instability due to hydrolysis. A recent report developed a unique solution to this limitation of electrophilic warheads by synthesizing and incorporating one or more hydrolysis-resistant, amine-reactive deoxyoxanosine (dOxa) nucleobases at the 3′ termini of aptamer sequences.^92,93,95^ This dOxa motif is capable of Watson–Crick base pairing with deoxycytidine (dC) and therefore was incorporated by exploiting the sequence similarity between dOxa and deoxyguanosine (dG). Therefore, the desired number of dC repeats were added at the 5′ terminus of the aptamer's cDNA, resulting in DNA polymerase-mediated addition of the equivalent number of dOxa repeats being added to the 3′ terminus of the aptamer. These terminal dOxa repeats are capable of crosslinking lysine residues on its target protein surface (Fig. 13A).

The authors proposed that the stabilized ring structure containing the carboxylic ester instilled structural stability and hydrolysis resistance. To test this, the same aptamers were appended with a terminal NHS ester or the dOxa moiety for comparison. The dOxa aptamer reported a 10^6^-fold increase in stability.

Proof of concept work was performed with two aptamers, TBA and HD22, which bind different sites on thrombin. Aptamer sequences containing 3–12 electrophiles were screened and the optimal numbers of 4 dOxa for TBA and 10 dOxa for HD22 were identified. Full crosslinking was observed using these aptamers with 1 eq. of TBA-dOxa and 2 eq. of HD22-dOxa after a 4–16 h incubation (Fig. 13B). Mass spec sequencing analysis revealed K440 (for TBA) and K537 (for HD22) as the predominantly modified residues, though additional labeling of K384 was observed with both aptamers and K440 with HD22. These lysine residues are positioned near the 3′ termini of the respective bound aptamer partner based on crystal structures (Fig. 13C).

The success of thrombin crosslinking was followed up by detection of the cell surface proteins PTK7 and nucleolin with their respective aptamers, sgc8c and AS1411. Each aptamer was appended with 10 dOxa repeats on its 3′ terminus,^95^ and >85% crosslinking of PTK7 and complete crosslinking of nucleolin was achieved with 4–5 equiv. of covalent aptamer and a 24 h incubation (Fig. 13D). The long incubation time was surprising, given the *t*_1/2_ of crosslinking for TBA and HD22 were 0.2 h and 1.7 h, respectively. Adding Cy5-modified cDNA to the two Cy3-labeled, covalently engaged aptamers at the cell surface enabled imaging of PTK7 and NLC (Fig. 13E). This revealed that PTK7 was localized at cell–cell interfaces along the apical membrane, while nucleolin was concentrated at the cell periphery on the basal membrane.

## 6. Aptamer-directed label transfer and protein crosslinking with *N*-acyl *N*-alkyl sulfonamides (NASA)

While the reports of covalent aptamers discussed thus far have solely consisted of nucleic acid-protein crosslinking, the use of cleavable electrophilic warheads allows for aptamer-mediated covalent label transfer of a small molecule to a protein of interest. The NASA electrophile is part of a suite of warheads used for ligand-directed (LD) label transfer, a method established by the Hamachi lab for the covalent labeling of endogenous proteins.^73,106–108,146^

**Fig. 14:**
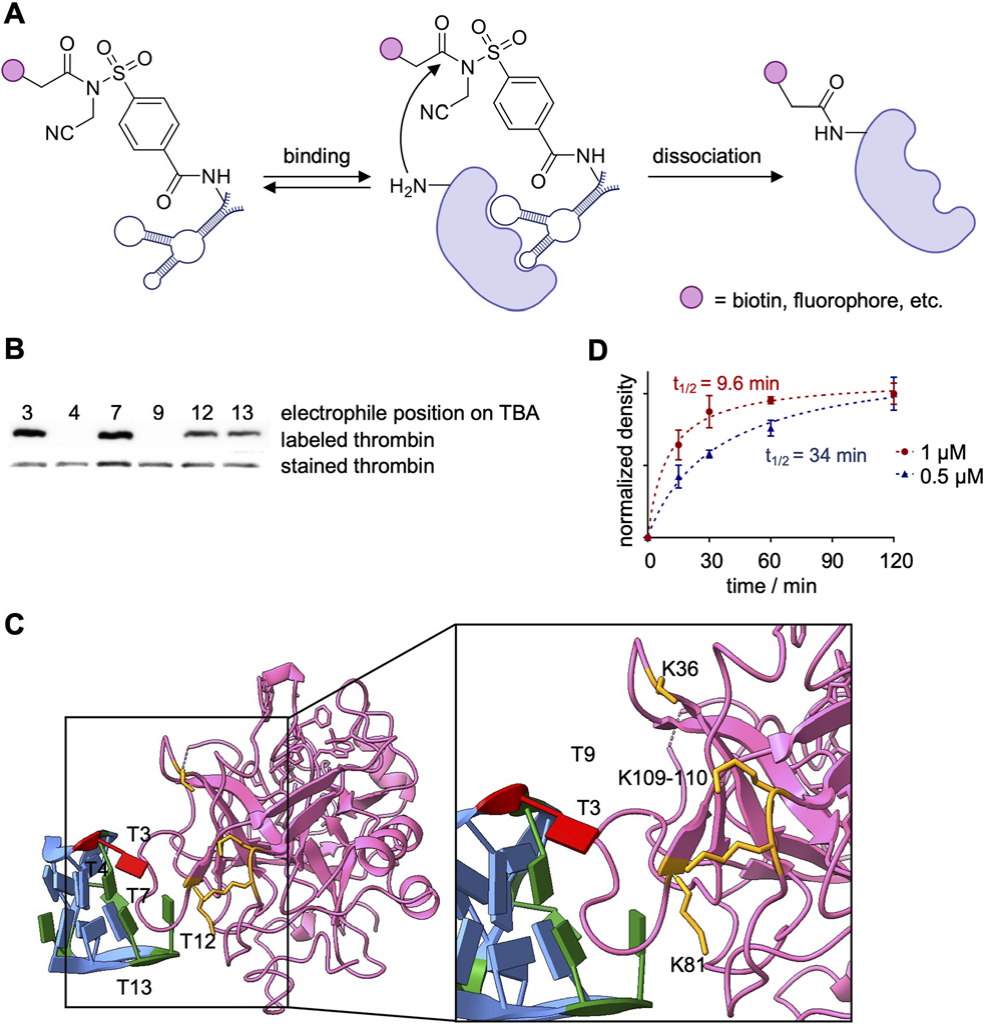
Covalent aptamer-mediated thrombin labeling. (A) Schematic depicting generation and POI labeling of NASA-functionalized aptamers. (B) Structure–activity relationship of the positional TBA isomers biotinylating thrombin. (C) Labeling kinetics of TBA with either 500 nM (blue) or 1 μM (red) of aptamer. (D) Crystal structure of TBA (light blue) bound to prothrombin (pink). Position 3 on TBA is highlighted red. Biotinylated residues are highlighted in orange (PDB 1HAO). Parts of this figure were adapted from ref. 147 with permission from John Wiley and Sons, copyright 2021.

The first use of NASA-functionalized aptamers, published by the Deiters lab, targeted thrombin as a proof-of-concept study. The same TBA sequence, later used by the Taki and Oh labs,^95,139,140^ was site-specifically equipped with a NASA electrophile through incorporation of an EdU nucleobase followed by functionalization through a Cu-catalyzed [3+2] cycloaddition.^147^ The cleavable electrophile, once brought in proximity to the protein surface by aptamer binding, covalently transfers a label onto a nearby lysine (Fig. 14A). A set of six TBA positional isomers was synthesized and a structure–activity relationship study revealed that TBA(3)-NASA, with the electrophile at position 3, showed the most efficient biotinylation of thrombin (Fig. 14B), TBA(7), TBA(12) and TBA(13) showed some labeling, while TBA(4) and TBA(9) showed no labeling. The lack of activity observed with TBA(9) was later confirmed by the Taki lab, as their TBA(9)-ASF showed little crosslinking activity, which can likely be attributed to TBA(9) positioning the electrophile distal to the aptamer–protein interface (Fig. 10C and 14C).^140,147^ While TBA(4) is located near the aptamer–protein interface, it is possible that no labeling is observed due to modification of this nucleobase potentially interrupting key binding interactions with R75 and R77 on thrombin.^141^ Interaction of these residues with TBA(13) could also cause the decreased labeling observed with that positional isomer.

The fast label transfer observed with TBA(3)-LDNASA (*t*_1/2_ = 34 min, Fig. 14D) outcompetes nuclease catalyzed degradation, which provides opportunities for future *in vivo* applications.^148,149^ Mass spec analysis determined that residues K149, K109, K110, and K36 were biotinylated, with K149 being the prime target (91%) (Fig. 14C). Unfortunately, K149 is not present in the crystal structure; however, the other residues, K109, K110, K81, and K36, are found along the interface of the protein–aptamer interaction. Labeling of these lysine residues is supported by the crystal structure, which shows the electrophile modification at TBA(3) oriented towards these residues (Fig. 14C). Additional mass spec sequencing of the residues labeled by the other positional isomers of TBA would provide insightful data towards the binding and labeling chemistry. Intact mass spec analysis revealed that 81% of thrombin molecules were biotinylated, with an average of 1.4 biotins per thrombin following incubation with 2 eq. of aptamer for 1 h. The biotinylated residues determined by the Deiters lab are positioned closely to the Y88 and H91 residues that were crosslinked by the TBA(3)-ASF, corroborating the crystal structure that shows the aptamer binds proximal to these residues.^140,147^

**Fig. 15:**
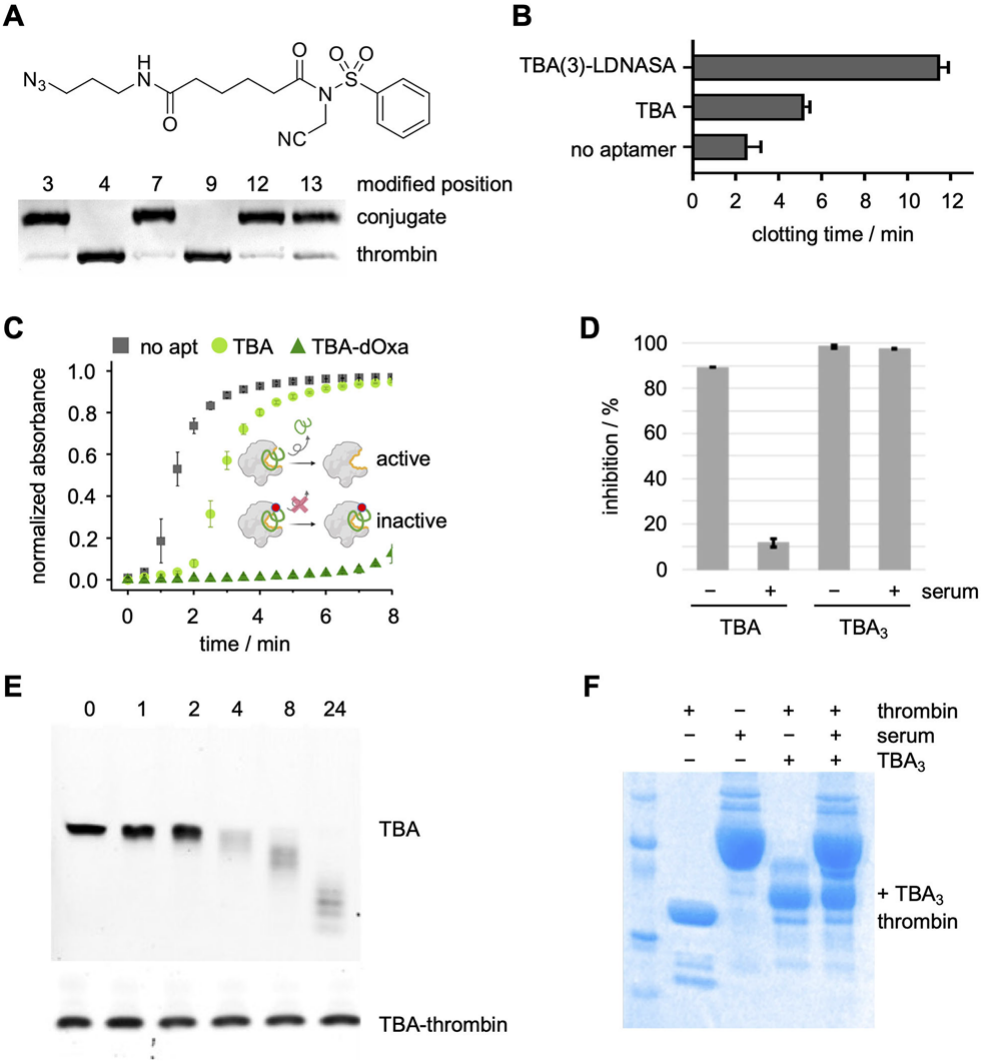
(A) Structure of the inverted NASA electrophile and SDS-PAGE analysis of TBA-NASA crosslinking to thrombin. (B)–(D) Inverted-NASA-, dOxa-, and ASF-aptamer enhanced inhibition of thrombin activity. (E) and (F) SDS-PAGE analysis of protein-crosslinked and non-crosslinked TBA stability in the presence of human serum and nucleases. Parts of this figure were adapted from ref. 95, 147, and 151 with permission from the American Chemical Society, copyright 2025, and John Wiley and Sons, copyright 2021.

The Deiters lab next inverted the NASA warhead for modification of the same TBA-EdUs, providing the ability to crosslink the nucleic acid ligand to thrombin (Fig. 15A).^147^ The six positional isomers showed a comparable SAR to the label-transferring aptamers, demonstrating generally applicable reactivity (Fig. 15A). Near complete crosslinking was observed after 1 h with TBA(3), TBA(7), TBA(12), and TBA(13), all forming a 1 : 1 aptamer–protein complex. To investigate the functional effect of covalent aptamer binding, a clotting assay of thrombin's proteolytic activity to convert fibrinogen to fibrin was performed.^150^ In this assay, the NASA-crosslinked aptamers improved inhibition of thrombin's enzymatic activity (12 min clotting time, Fig. 15B) compared to the unmodified aptamer (6 min).^147^ Similar assays were also performed by the Taki and Oh labs with their ASF-modified and dOxa-modified TBA aptamers to inhibit thrombin, showing similar trends (Fig. 15C).^95,140^ Importantly, the Taki lab showcased the maintained ability of the crosslinked-TBA to inhibit thrombin in the presence of nucleases, while unmodified TBA was unable to do so (Fig. 15D).^140^

While this instability of aptamers towards nucleases has traditionally been addressed with chemical modifications,^6,54–56^ these studies suggest that crosslinking of aptamer to its protein target could instill increased stability towards nuclease activity. First, the Deiters lab assessed the stability of their aptamer–thrombin complexes towards nuclease-mediated degradation in human serum.^147^ The NASA-mediated crosslinked aptamer–protein complex maintained integrity for over 24 hours in human plasma, whereas non-crosslinking aptamer was degraded in just 2 hours under the same conditions (Fig. 15E). These results were independently confirmed by the Taki lab, who also showed stability of the covalent thrombin–aptamer complexes to recombinant nucleases for 24 hours (Fig. 15F).^151^ Thus, covalent aptamers could facilitate the clinical translation of aptamers as therapeutic options should the unexpected stability of crosslinked aptamer–protein complexes be a general phenomenon.

**Fig. 16:**
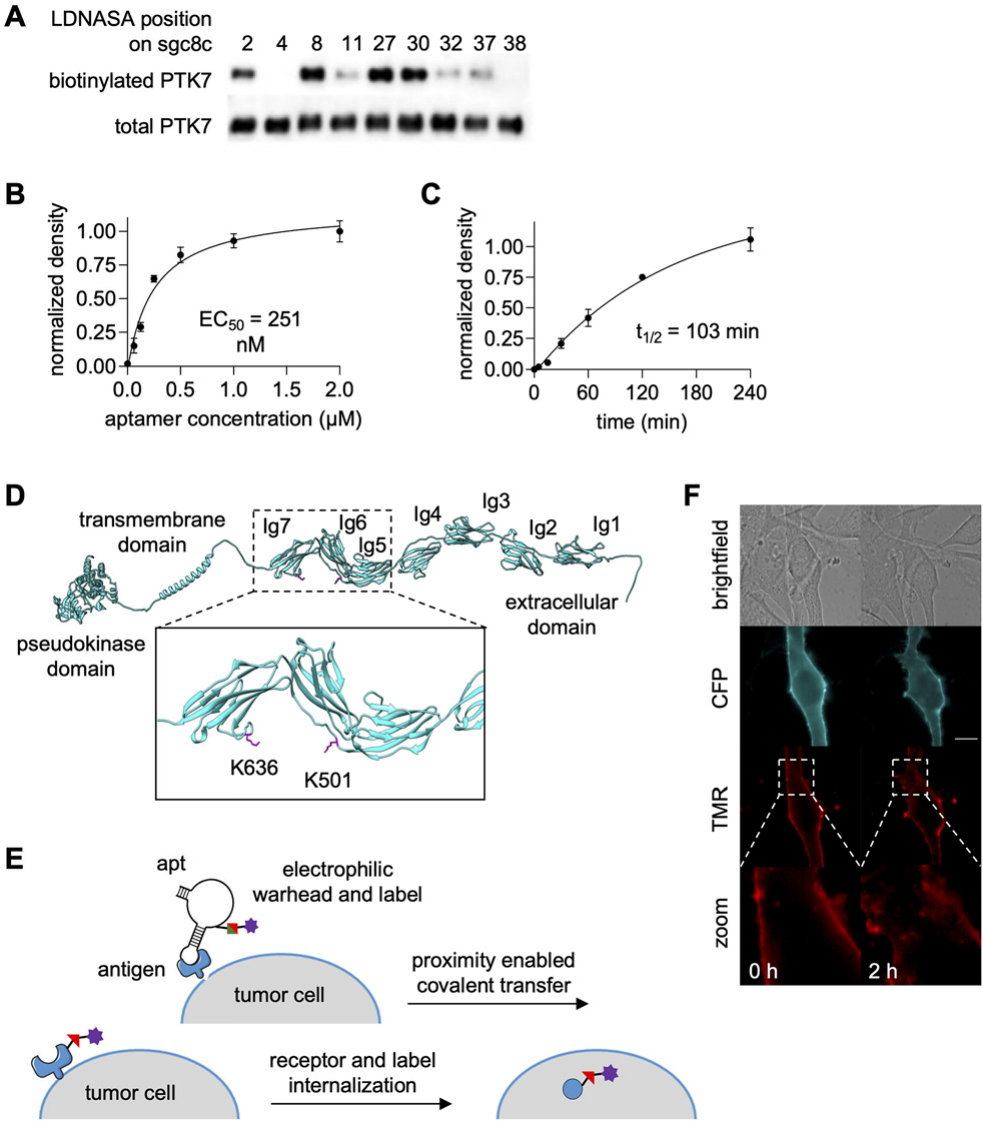
Covalent aptamer-mediated PTK7 labeling. (A) Position-dependent reactivity of sgc8c-NASA mediated PTK7 labeling. (B) EC_50_ determination and (C) *t*_1/2_ of label transfer to PTK7 expressed on HEK293T cell surfaces. (D) Mass spec analysis of labeled residues on PTK7. (E) Schematic of PTK7 labeling and internalization. (F) Endosomal internalization of SA-TMR on biotinylated cells surfaces. Parts of this figure were adapted from ref. 152.

The next implementation of NASA-modified aptamers targeted the cell-surface expressed cancer biomarker PTK7. The PTK7-targeting aptamer sgc8c was site-specifically modified with OdU nucleobases for conjugation to the NASA-biotin electrophile.^152^ SAR studies revealed that position 27 afforded the most efficient biotin transfer and was therefore chosen for further study (Fig. 16A). Similar EC_50_ values were observed for both recombinant protein labeling and labeling of PTK7 on cell surfaces along with a *t*_1/2_ of 103 minutes (Fig. 16B and C), showing promise for biological applications that require overcoming nuclease susceptibility. Mass spec sequencing showed biotinylation of residues K501 and K636 (Fig. 16D). While the binding site of sgc8c on PTK7 is yet to be determined, this data suggests it to be near the Ig6 and Ig7 extracellular domains. Aptamer-mediated biotinylation of cancer cell surfaces was used to recruit a streptavidin-small molecule conjugate, specifically SA-TMR (tetramethyl rhodamine). Internalization of SA-TMR to endosomes was observed two hours post-labeling, indicating promise for targeted drug delivery (Fig. 16E and F).^152^ While this exciting proof-of-concept work has showcased highly efficient biotinylation of a cell surface biomarker, this work can and should be expanded to other cell surface targets with more biologically relevant small molecule labels.

**Fig. 17:**
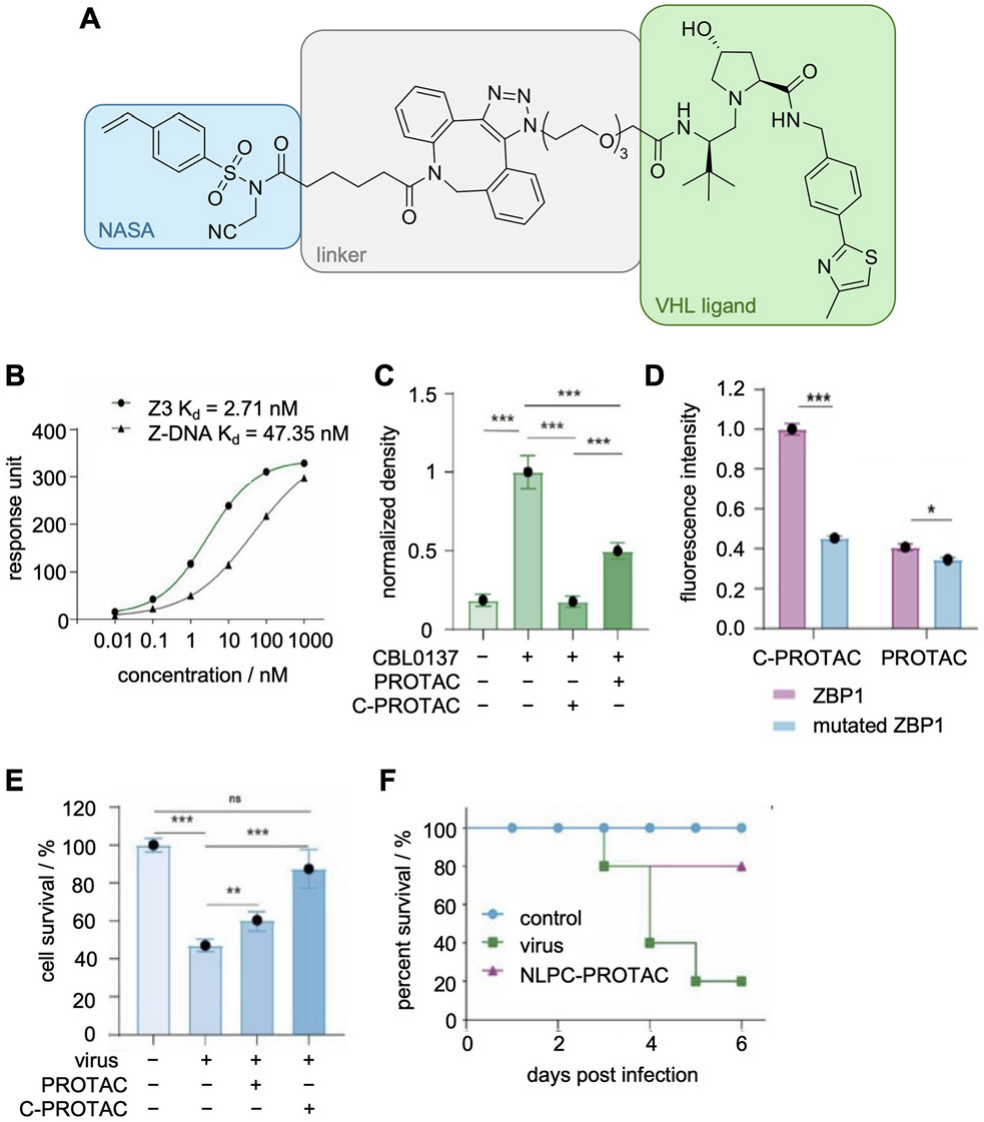
Aptamer-medicated covalent transfer of VHL ligands for the degradation of target proteins. (A) Structure of NASA-VHL electrophile. (B) SPR analysis of aptamer Z3. (C) Degradation of ZBP1 as measured by western blot. (D) Comparison of relative fluorescence intensity of wild-type or mutated ZBP1 incubated with Z3-NASA-Cy3 (mimicking C-PROTAC) or Z3-Cy3 (mimicking PROTAC). (E) Survival of H1N1-infected cells treated with the indicated aptamer. (F) Survival of infected mice treated with nanoliposome containing covalent aptamer. Parts of this figure were reproduced from ref. 155 from John Wiley and Sons, copyright 2025.

ApTACs, aptamers modified with E3-recruiting ligands, are capable of inducing degradation of the target protein, similar to a PROTAC.^153,154^ More recently, Huang, *et al.* synthesized a NASA electrophile capable of transferring a Von Hippel–Lindau (VHL) ligand to recruit E3 ligands to a covalently modified protein of interest (Fig. 17A).^155,156^ They opted to target the Z-DNA binding protein 1 (ZBP1) due to its role in viral infection and performed a traditional SELEX selection to generate their new ZBP1-targeting aptamers.^157–159^ The aptamer library was structured based on the consensus Z-DNA conformation that ZBP1 is known to bind and resulted in 5 potential aptamers that were further characterized *via* SPR. The best aptamer had a *K*_D_ of 2 nM, a 20-fold increase in affinity compared to the consensus Z-DNA sequence (*K*_D_ = 48 nM, Fig. 17B), and was functionalized with a terminal DBCO to install an azido carrying NASA-VHL. To showcase the ability to degrade ZBP1 on cells, the protein was upregulated with CBL0137, a small molecule that induces Z-DNA formation in cells, and subsequently treated with either their covalent aptamer or an analog conjugated to the VHL ligand with no electrophile present.^160^ While the non-covalent aptamer did induce some degradation of ZBP1, there was a 3-fold increase in degradation when the VHL ligand was covalently transferred to ZBP1 (Fig. 17C). Covalent transfer was confirmed by mutating nucleophilic amino acids in the ZBP1 sequence, which reduced label transfer (Fig. 17D). The authors further studied the downstream signaling effects due to ZBP1 degradation and its positive impact on the survival of cells infected with the H1N1 virus (Fig. 17E).

For *in vivo* experiments, the covalent aptamer was encapsulated in liposomes for delivery and administered to mice with an induced inflammatory response. The nanoliposome was stable in mice for 72 h, an impressive feat compared to the 1 h half live of aptamers in serum.^148,149^ Treated mice showed improved survival toward infection (Fig. 17F), demonstrating the potential of a new class of antiviral agents using aptamers as targeting agents for infected cells.

Outside of aptamers, an RNA ligand based off of the RNA consensus binding element of an RBP was functionalized with a NASA electrophile capable of transferring a hydrophobic tag to its cognate binding proteins, Lin28a, leading to degradation of this protein.^138^ As Lin28a inhibits productions of let-7 miRNA, there was a subsequent increase in these mature miRNAs, which act as tumor suppressors.^161^

## 7. Aptamer-directed attempts at catalytic protein labeling

**Fig. 18:**
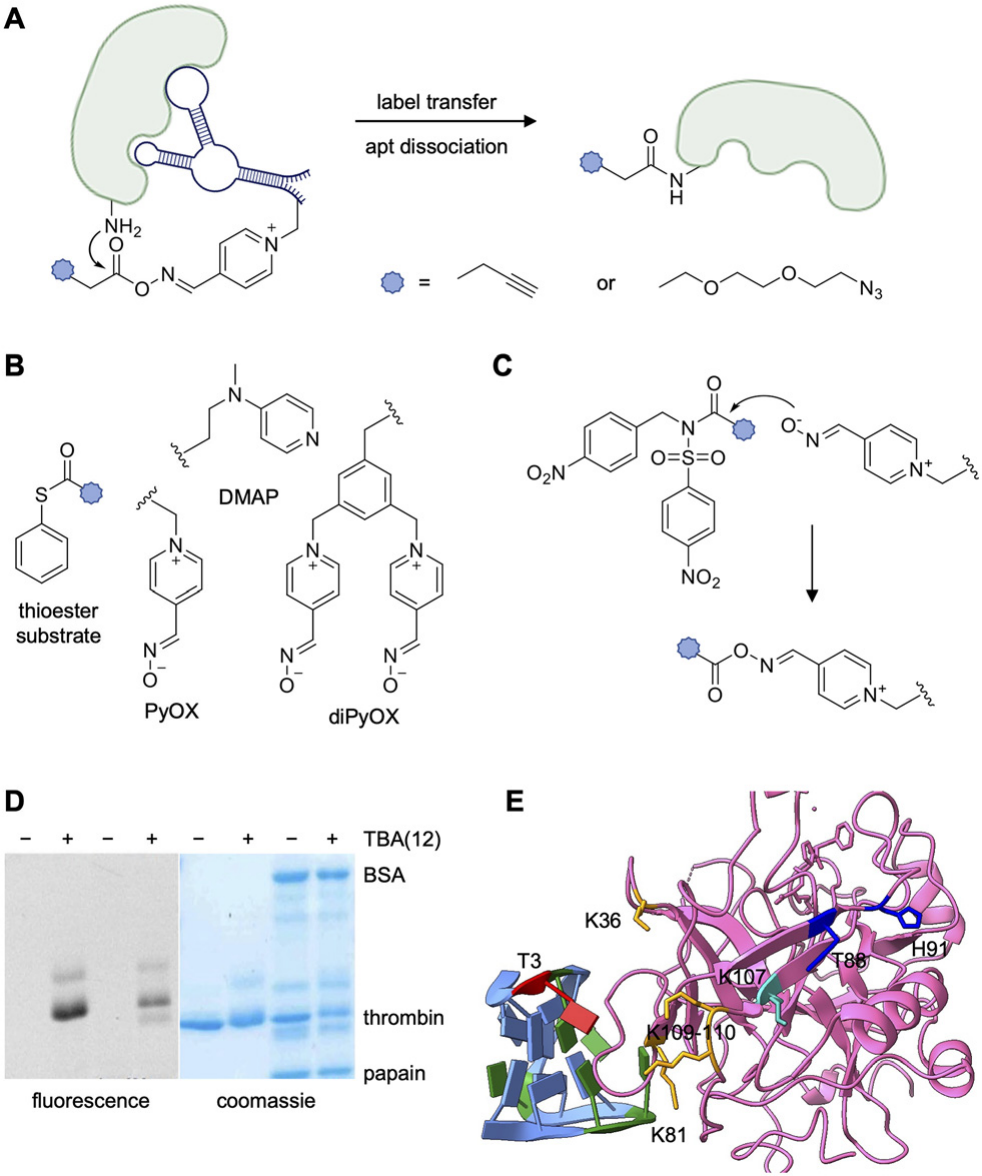
Aptamer-mediated catalytic labeling of thrombin. (A) Mechanism of PyOx catalyst forming charged intermediate for subsequent label transfer (B) Schematic of catalyst-modified aptamer binding to its target protein and proximity-induced label transfer. (C) Structure of synthesized catalysts and substrates. SDS-PAGE analysis of the specificity of (D) TBA(12)diPyOX for thrombin when incubated in a complex mixture of off-target proteins. Results visualized by in-gel fluorescence and Coomassie stain. (E) Crystal structure of TBA (light blue) complexed with prothrombin (pink). Position 3 on TBA is highlighted red. Biotinylated residues as determined by mass spec analysis are highlighted orange (TBA(3)-LDNASA), blue (TBA(3)-ASF), or cyan (TBA(3)-diPyOx) (PDB 1HAO). Parts of this figure were reproduced from ref. 163.

Two labs designed systems for catalyzing covalent label transfer from aptamer to target protein. As opposed to tethering an electrophile to the oligonucleotide itself, the Albada lab functionalized TBA with organocatalysts. Using a system inspired by foundational work of the Hamachi lab,^162^ the group tested their aptamer-mediated workflow with two acyl-transfer catalysts, 4-(*N*,*N*-dimethylamino)pyridine (DMAP) and 4-pyridinecarbaldehyde oxime (PyOx) (Fig. 18A and B).^163^ Organocatalysts were synthesized with azide handles and attached at individual alkyne-modified thymidine residues in the TBA sequence *via* CuAAC reactions. The “clicked” catalyst activated an acyl donor substrate as a charged intermediate with increased reactivity, which in turn is attacked by proximal nucleophilic residue on the bound protein (Fig. 18C).^163^ Thioester and NASA substrates are activated by DMAP and PyOx, respectively. The organocatalyst–aptamer complex can repeat the activation process with another substrate molecule or can dissociate and bind another protein. This would, in theory, enable lower aptamer concentrations, decreasing the likelihood of off-target labeling.

Both TBA(3)-DMAP and TBA(12)-DMAP were synthesized and first incubated with thrombin followed by addition of the azido-thioester substrate. Protein labeling was visualized by gel shift after conjugation of BCN-PEG2000 and modification of 27–49% of thrombin was observed. However, up to 20% of thrombin labeling was the result of simple background reaction between the target protein from the azido-thioester substrate. Despite the background activation, catalyst-clicked aptamers showed specific fluorophore transfer to thrombin in the presence of multiple off-target proteins, which suggests that the presence of the aptamer directs the activated thioester towards thrombin faster than the background reaction occurs. However, a large excess of catalyst (3 eq.) was needed for efficient labeling, rendering the reaction non-catalytic.

While there was no decrease in background labeling with the PyOX catalyst paired with a NASA electrophile, labeling efficiency was further reduced (18–28%). To counteract this, aptamers functionalized with two PyOX catalysts, instead of one, were assembled, which resulted in near quantitative labeling of thrombin (93%) in the presence of multiple off-target proteins (Fig. 18D). Mass spec sequencing showed that K106 for TBA(3)-diPyOX and K12 and K77 for TBA(12)-diPyOX were the primary targets, indicating the positioning of the catalyst on the aptamer directly influences which lysine residues on the target is labeled.^163^ While the Taki lab (Y88 and H91, blue), Deiters lab (K36, K109–110, and K149, orange), and Albada lab (K106, red) all used TBA modified with an electrophile at position 3, the labeling of different residues on thrombin was observed. However, all were positioned along the same surface of the protein (Fig. 18D).^140,147,163^ This could be due to the different size of the warheads and linker designs, or a result of the different covalent chemistries. Regardless, the potential for background labeling in the absence of the catalyst and the necessity to use an excess of the catalyst-aptamer conjugate leaves room for further optimization.

**Fig. 19:**
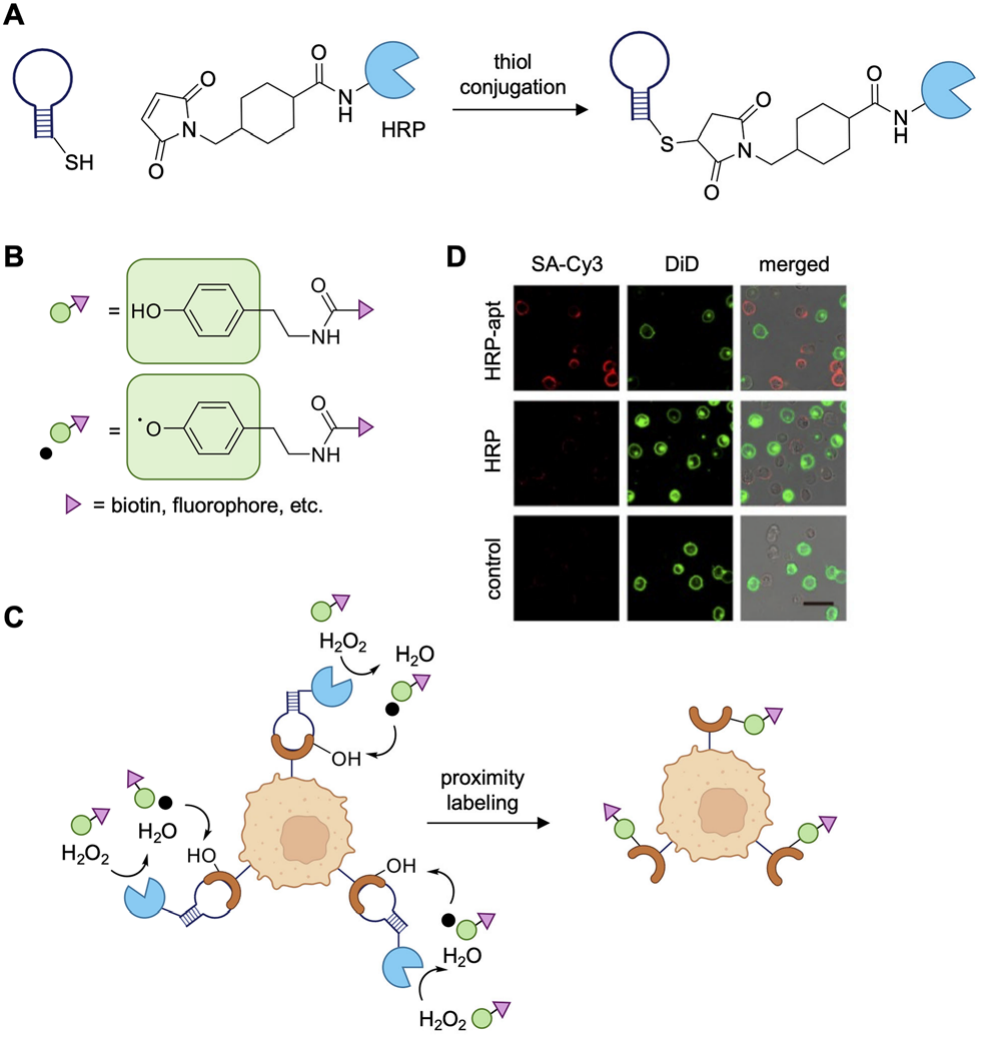
Radical labeling of tyrosine residues. (A) Schematic of aptamer-HRP synthesis through a maleimidomethyl-NHS-ester bifunctional linker. (B) Structure of phenol-label and corresponding radical formation. (C) Schematic of horseradish peroxidase-aptamer (HRP-apt) induced covalent labeling of tumor cells. (D) Targeted labeling of mixed cell population (target: HL60, non-target: K562 cells, pre-stained with green cell membrane dye DiD). BP labeling visualized with SA-Cy3 (red). Scale bars: 20 μm. Parts of this figure were reproduced from ref. 164 with permission from American Chemical Society, copyright 2023.

The Ju lab also developed an aptamer-based, proximity-enabled catalytic labeling approach, utilizing a design that resembles APEX and related methodologies.^164^ Here, the aptamers KH1C12 and T2-KK1B10, which target the HL60 and K562 leukemia cell lines, respectively, were conjugated to horse radish peroxidase (HRP) using a maleimidomethyl-NHS-ester bifunctional linker (Fig. 19A). When incubated with biotin-phenol (BP) in the presence of hydrogen peroxide (H_2_O_2_), HRP catalyzes the formation of BP hydroxyl radicals, which undergo covalent bond formation with protein surface residues (Fig. 19B). The catalytic aptamers were evaluated for specificity in mixed cell populations to show labeling of only the appropriate target leukemia cell line (Fig. 19C), where the short lifetime of the radical intermediates ensure cellular specificity. Quantification of labeling showed that over 97% of target cells were labeled with 95% accuracy. However, mass spec analysis of the identity of target proteins was not performed, which would have provided detailed information on the aptamer interaction partners.^164^

## 8. Summary

Covalent protein–nucleic acid complexes have previously been reviewed in the context of structural analyses^165^ and as covalent tags for protein functionalization.^166^ Here, we specifically summarized the recently emerging modification of aptamers with electrophilic warheads, which holds promise in diagnostic and therapeutic applications due to the synthetic accessibility and ease of chemical modification, while retaining the high specificity and affinity of aptamers towards their protein targets. Aptamers offer opportunities to target proteins that have traditionally been considered “undruggable” by small molecule ligands. Additionally, compared to their antibody counterparts, aptamers lack strongly nucleophilic sites, enabling the installation of select electrophilic warheads. For instance, the pseudokinase PTK7 does not have a known small molecule ligand, but its aptamer has seen widespread applications, *e.g.*, as an imaging and drug delivery agent.^23,46,47,152^ Both crosslinking and label transferring aptamers have been utilized for biological applications, including directed cargo internalization, sensitive detection of specific cells, and inhibition of enzyme activity.^95,140,147^ Thus far, covalent aptamers have been developed targeting a variety of proteins including PDGF-BB, thrombin, PTK7, S-protein, streptavidin, mAbs, TfR1, nucleolin, and ZPB1, and some targeting whole tumor cells, including DLD-1, HCT116, HL60, and K562 cells. While the vast majority of covalent aptamers were created from literature-reported non-covalent counterparts, dedicated SELEX approaches for the *de novo* selection of covalent aptamers have been reported as well.^57,129^

In nearly all examples, the addition of covalency enhanced the functionality of covalent aptamers compared to their non-covalent counterparts, including an increased stability of the aptamer to nucleases, overcoming one of the major hurdles for therapeutic success. For instance, accumulation of covalent aptamers was observed at tumor sites in mice despite renal filtration and/or degradation of its non-covalent counterpart.^114^ Additionally, multiple labs showcased an increased stability of aptamers towards nuclease degradation when covalently crosslinked to their target protein.^147,151^ Thus, the covalency has led to improved pharmacokinetic properties with promise for increased *in vivo* efficacy.

Covalent aptamers have the potential to be further developed into therapeutic agents for targeted drug-delivery, immune cell recruitment, protein degradation, among many other applications. For example, covalent aptamers targeting the S-protein and the RBD domain of SARS-CoV-2 were developed and showed increased detection sensitivity and enhanced virus neutralization compared to their non-covalent analogs.^57,127^ Moreover, aptamer crosslinking or transfer of ligands has been utilized for targeted protein degradation, leading to cancer cell killing and a reduction of tumor burden in mouse xenografts.^143,155^

Covalent engagement of the target protein effectively sets *k*_off_ rates to zero, eliciting more pronounced biological effects. The reports of electrophile-modified aptamers summarized here demonstrate quick kinetics, high labeling yields, and broad applicability, although there is still room for further improvement. The majority of the reported covalent aptamers have been DNA aptamers, but with careful electrophile selection this technology could be expanded to RNA aptamers, keeping in mind previous studies that have utilized electrophiles for selective and covalent modification of RNA.^65–69^ As RNA tends to be more prone to degradation, covalent bond formation between the RNA and protein of interest will theoretically increase the stability of these nucleic acid probes. RNA is capable of folding into more complex three-dimensional structures than DNA, which could facilitate the discovery of more covalent aptamer–protein pairs. Additionally, some studies discussed in this review did not report mass spec analysis of the modified protein. While few protein–aptamer structures have been elucidated, label transfer offers a unique ability to map interaction sites, as shown in the example of PTK7. For aptamers that have unknown target proteins since they were discovered by cell-SELEX processes, covalent bond formation could play an integral role in target identification *via* mass spec sequencing.^167^ There are several additional electrophiles that should be explored, such as recently reported dibromophenyl benzoates, benzotriazoles, and *N*-acyl-*N*-aryl sulfonamides.^106,108,110^ These electrophiles have increased reactivity and/or stability compared to other warheads and have the potential to increase the efficiency or specificity of aptamer-mediated protein modification. It is important to point out that higher reactivity of the electrophile could result in covalent bond formation to off-target proteins, amplifying negative side effects that may not be observed with non-covalent counterparts. For this reason, it is of the utmost importance and interest to develop and utilize aptamers with specificity that rivals that of small molecule covalent drugs. The off-target toxicities of covalent small molecule inhibitors have been mitigated through the use of reversible covalent inhibitors, which is another reactive motif that could be used here as well.^168^

While outside the scope of this review, covalent bonds between aptamers and proteins have been establish using photocrosslinkers, such as azides,^169,170^ diazirines,^171,172^ and 5-iodo- or 5-bromo-2′-deoxyuridines.^173–175^ In addition, photocrosslinking has been integrated into the SELEX process,^174^ suggesting the ability to develop new aptamers to replace those that cannot be modified with photoreactive groups.

Looking ahead, the continued development of covalent aptamers will benefit from systematic improvements in electrophile selection and SELEX methodologies. Key priorities include optimizing the balance between reactivity and selectivity to minimize off-target effects, expanding the use of mass spectrometry for comprehensive target characterization, and conducting thorough pharmacokinetic studies in relevant disease models. As more structure–activity relationships are established, covalent aptamers may prove useful for targeting proteins that remain challenging for conventional small molecules and antibodies. While significant questions remain regarding their clinical translation, the combination of aptamer specificity with covalent engagement offers a distinct approach that warrants further investigation across therapeutic and diagnostic applications.

## Conflicts of interest

There are no conflicts to declare.

## Data Availability

No primary research results, software or code have been included and no new data were generated or analyzed as part of this review.
